# Transcriptome profile of the early stages of breast cancer tumoral spheroids

**DOI:** 10.1038/srep23373

**Published:** 2016-03-29

**Authors:** Rosario Pacheco-Marín, Jorge Melendez-Zajgla, Gonzalo Castillo-Rojas, Edna Mandujano-Tinoco, Alfredo Garcia-Venzor, Salvador Uribe-Carvajal, Alfredo Cabrera-Orefice, Carolina Gonzalez-Torres, Javier Gaytan-Cervantes, Irma B. Mitre-Aguilar, Vilma Maldonado

**Affiliations:** 1 Epigenetics, National Institute of Genomic Medicine, Periférico Sur No. 4809, Col Arenal Tepepan, Delegación Tlalpan, México, D.F., C.P 14610; 2 Functional Genomics laboratories, National Institute of Genomic Medicine, Periférico Sur No. 4809, Col Arenal Tepepan, Delegación Tlalpan, México, D.F., C.P 14610; 3 Microbial Molecular Immunology Program, Department of Microbiology and Parasitology, Faculty of Medicine, National Autonomous University of Mexico (UNAM), University City Avenue 3000 C.P. 04510, Coyoacan, Mexico City; 4 Department of Molecular Genetics, Institute of Cellular Physiology (UNAM), University City Avenue 3000 C.P. 04510, Coyoacan, Mexico City; 5 Posgraduate Program in Biological Sciences, Faculty of Medicine (UNAM), University City Avenue 3000 C.P. 04510, Coyoacan, Mexico City; 6 Unit of Biochemistry, National Institute of Medical Sciences and Nutrition Salvador Zubirán (INCMNSZ), Av. Vasco de Quiroga N° 15, Colonia Belisario Domínguez Sección XVI, Delegación Tlalpan. CP.14080, México D. F., México

## Abstract

Oxygen or nutrient deprivation of early stage tumoral spheroids can be used to reliably mimic the initial growth of primary and metastatic cancer cells. However, cancer cell growth during the initial stages has not been fully explored using a genome-wide approach. Thus, in the present study, we investigated the transcriptome of breast cancer cells during the initial stages of tumoral growth using RNAseq in a model of Multicellular Tumor Spheroids (MTS). Network analyses showed that a metastatic signature was enriched as several adhesion molecules were deregulated, including EPCAM, E-cadherin, integrins and syndecans, which were further supported by an increase in cell migration. Interestingly, we also found that the cancer cells at this stage of growth exhibited a paradoxical hyperactivation of oxidative mitochondrial metabolism. In addition, we found a large number of regulated (long non coding RNA) lncRNAs, several of which were co-regulated with neighboring genes. The regulatory role of some of these lncRNAs on mRNA expression was demonstrated with gain of function assays. This is the first report of an early-stage MTS transcriptome, which not only reveals a complex expression landscape, but points toward an important contribution of long non-coding RNAs in the final phenotype of three-dimensional cellular models.

Multicellular tumor spheroids (MTS) are a type of cell culture that recreates the microenvironmental, molecular, functional and histomorphological characteristics of *in vivo* tumors. MTS acquire differentiated cell-cell junctions and a defined microenvironment. Later, the MTS grow to diameters between 200 and 500 μM, form defined gradients of nutrients and oxygen, and establish a central apoptotic/necrotic area as they reach sizes larger than 500 μM^1^. The initial growth phase of the MTS is reminiscent of the small avascular growth during the initial growth of primary tumor and metastasized cells. The cell-cell interactions and microenvironment of these structures results in multicellular resistance, a well-defined phenotypic change that is also present in tumors *in vivo*^2^, and causes enhanced invasion and migration, as well as an increased clonogenic capacity. Although earlier studies have reported that phenotypic changes in MTS are associated with modulation of cellular transcription, relatively few studies have been conducted that analyze gene expression in MTS. Francia *et al.*^3^ performed differential display to find genes regulated in MTS and identified a network of expression that suppressed DNA mismatch repair and was responsible for multicellular resistance to alkylating agents^3^. Similarly, Zhou *et al.*^4^ used dynamic array device integrating microfluidic circuits to analyze gene expression in MTS and found changes in cellular senescence and glucose metabolism genes^4^. Nevertheless, there are no genome-wide analyses to date characterizing early stage MTS.

MTS model is frequently used to analyze the effectiveness of anticancer drugs because for many chemotherapeutic drugs this model provides a well-established solid tissue environment that is associated with multicellular resistance, which is a better reflection of *in vivo* conditions^5^,^6^.

Long non-coding RNAs (lncRNAs) belong to a group of molecules without protein coding potential that range widely in size, from 200 to 10000 nucleotides. Similar to messenger RNAs, lncRNAs are transcribed by RNA polymerase II, 5′ capped, spliced and polyadenylated at the 3′ end^7^. LncRNAs play a pivotal role in the regulation of gene expression at several cellular levels, including the organization of nuclear sub-structures, the modulation of chromatin states, transcription and post-transcriptional processing^8^. In addition, lncRNAs can function as co-factors of proteins, messenger RNA stabilizers, miRNAs regulators, or translation modulators^9^,^10^. The prevalence of lncRNAs is a subject of current debate, with large differences found depending in the technique used to identify them, and the particular definition used for their characterization. For example, Kaessmann H, *et al.* in 2014 identified all the lncRNAs of 11 tetrapod species and found 11000 lncRNAs in primates (almost 15000 in human) with 2500 of them highly conserved^11^. Given the recent identification of most lncRNAs, and their sheer number, their specific molecular functions remain unknown. Nevertheless, the importance of lncRNAs can be inferred due to their tissue-specific expression and specific regulation in diverse cellular processes, in both physiological and pathological conditions, such as genomic imprinting^12^, development^13^, pluripotency maintenance^14^, neurodegenerative disorders^15^, cardiovascular diseases^16^ and cancer^17^.

To gain more insight into the involvement of lncRNAs and mRNA in the cancer-associated phenotype at the early stages of tumoral and metastatic growth, we used small MTS with a size of <200 microns, which exhibit the classical multicellular resistance phenotype, to perform whole-genome transcriptome analyses.

## Results

### Characterization of MTS

**Figure 1:**
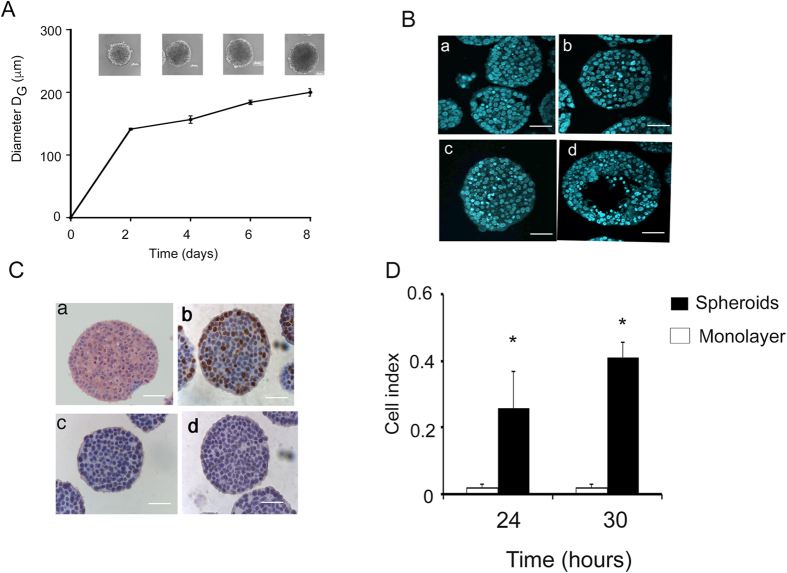
Growth and characterization of MTS. (**A**) Growth kinetics of MTS at 2, 4, 6 and 8 days of culture. ±SD n = 3, (**B**) Nuclei of cells of MTS at 2, 4, 6 and 8 days stained with DAPI (panel a, b, c and d respectively). (**C**) Six-day spheroids stained with H&E (a); and immunostaining with Ki67 (b); p27Kip1 (c); HIF1α (d). (**D**) Migration data monolayers vsspheroids disaggregated. The mean geometric diameter D_G_. Scale bar = 50 μm.

We were interested in analyzing the MTS that most closely mimic *in vivo* avascular tumors, because these MTS more accurately reflect the initial steps of cancer and micro-metastasis. For this, we initially characterized MTS cultivated during a eight-day course. As shown in Fig. 1A, MTS cultured for six days exhibited diameters of ±200 μM, with only a small percentage of condensate (apoptotic) nuclei, similar to day 2 and 4 (Fig. 1B, panel a–c). This is consistent with previous reports that showed that hypoxic conditions generally start in the central areas of MTS when approaching 200–250 microns in diameter^1^. In some MTS, this stratification was evident at 8 days, as shown in Fig. 1B, panel d. To further characterize the functional dynamics of the MTS, we quantified cell proliferation using Ki67 immunocytochemistry. Ki67 is a nuclear protein that is present during all active phases of the cell cycle (G1, S, G2 and mitosis) but is absent from resting cells (G0). Figure 1C, panel b, shows that approximately 40% of the cells were positive for this nuclear protein. In addition, we found that approximately 60% of cells were positive for the cyclin-dependent kinase inhibitor 1B (p27Kip1) (Fig. 1C panel c), an enzyme inhibitor that regulates G0-to-S-phase transition.

As expected, at day 6 we could not identify expression of hypoxia-inducible factor 1 (HIF1α), the main transcription factor that regulates hypoxia (Fig. 1C, panel d). In conclusion, we found that, at six days, MTS were already well-consolidated, in contrast to the previous days, during which the MTS were just cell aggregates that dislodge easily. At this early timepoint there is no stratification, no hypoxic areas, and more importantly, no evidence of cell death at the center of the MTS, in contrast to later timepoints. For these reasons, we selected the six-day timepoint for all our experiments.

### Migration ability of cells grown as MTS

Since active migration of tumor cells is a prerequisite for metastasis, we evaluated this ability in cells derived from MTS using the xCELLigence technique. As shown in Fig. 1D, MCF7 cells grown as MTS increased their ability to migrate by 10 times, when compared with cells grown as monolayer cultures (Table S2).

### Genome-wide expression analysis

To gain insight into the non-coding and coding RNA transcriptome that drives the early MTS culture, we performed a differential expression analysis using RNASeq in six-day MTS versus monolayer cultures.

### Coding RNA

**Figure 2:**
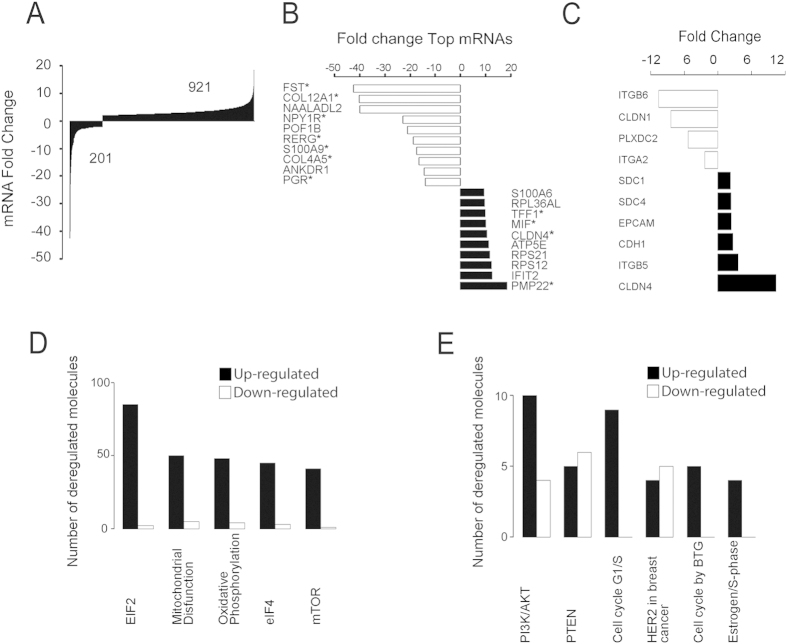
Analysis of the differentially expressed mRNAs in MTS using RNA sequencing. (**A**) Fold change of all mRNAs dysregulated. (**B**) Top ten of dysregulated mRNAs analyzed with IPA, * Genes related to breast cancer metastasis. (**C**) Fold change of mRNAs associated with cell adhesion. (**D**) Top five canonical pathways and (**E**) pathways associated with cell cycle using the IPA software.

In our analysis, we found 1122 dysregulated transcripts. Nine-hundred and twenty-one transcripts increased their expression; we found that 201 transcripts were regulated down-regulated and three of them (ANKRD30A, HBB and TCN1) were expressed only in monolayer cultures (Fig. 2A)

In Fig. 2B we show the top 20 (up and down) regulated genes. All them were associated with breast cancer and eleven have been associated with breast cancer metastasis (Table S3).

Interestingly, we found important differences in a group of 10 mRNAs that code for membrane proteins, which could be a reflection of the MTS consolidation at this stage. For example, we detected a robust decrease in Claudin 1 (CLDN1), Plexin 2 (PLXDC2), Integrin beta 6 (ITGB6), Integrin alpha 2 (ITGA2) and an increase in E-cadherin (CDH1), Integrin beta 5 (ITGB5), Claudin 4 (CLDN4), EPCAM and transmembrane proteoglycans such as syndecans (SDC1 and SDC4) in MTS (Fig. 2C).

To gain a global perspective on the main cellular processes modulated, we analyzed our data using Ingenuity Pathway Analysis (IPA) software. Using this tool, we found several dysregulated networks, with the top five dysregulated networks being Mitochondrial Dysfunction, Oxidative Phosphorylation, EIF2 Signaling, Regulation of elF4 and p70S6K and mTOR Signaling (Fig. 2D, Table S4 and Fig. S2). Additionally, we identified some interesting pathways associated with the cell cycle, such as estrogen-mediated S-phase entry, HER-2 signaling in breast cancer, cell cycle G1/S checkpoint regulation, cell cycle regulation by BTG family protein, PI3K/AKT and PTEN signaling (Fig. 2E). Supplementary Table S5 shows the up and down regulated molecules associated with cell cycle pathways, and an image of the canonical pathways (Fig. S3). Some other canonical pathways related to other cellular processes are also included, such as cell survival, cell adhesion and ubiquitination (Fig. S4).

**Figure 3:**
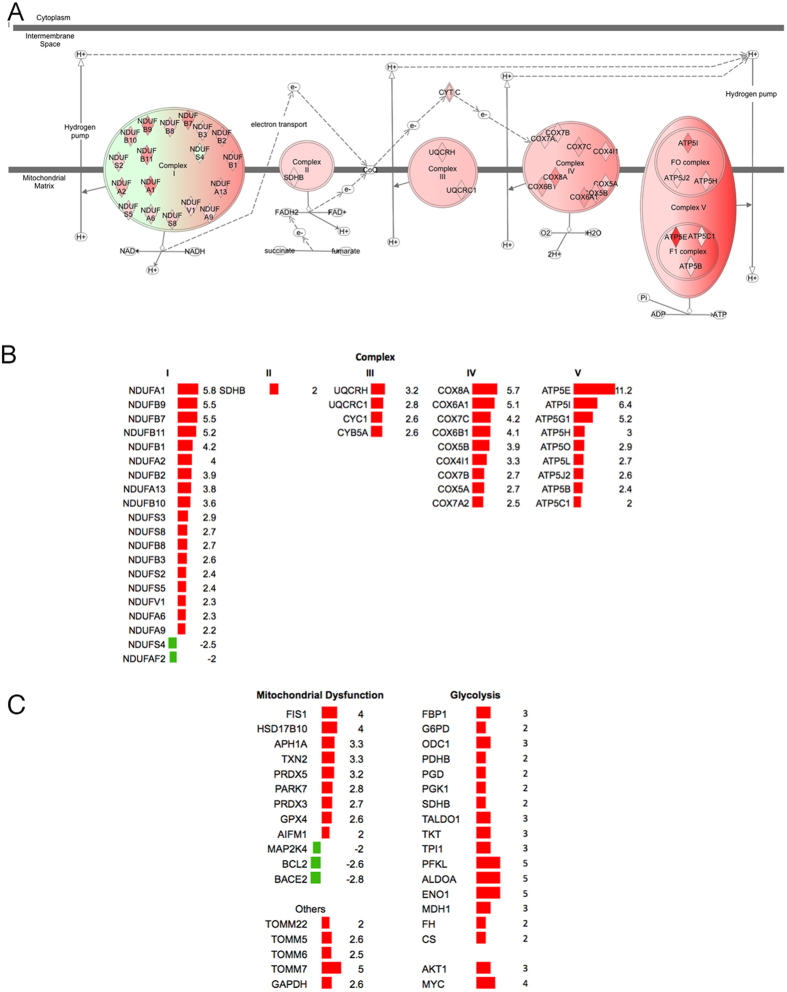
Hyperactivation of oxidative mitochondrial metabolism in MTS. (**A**) mitochondrial mRNAs dysregulated in five oxidative phosphorylation complexes. (**B**) Fold change of mRNAs dysregulated in the five oxidative phosphorylation complexes. (**C**) Fold change of mRNAs mitochondrial dysfunction proteins and other proteins and glycolysis genes. Red color = up-regulation and green color = down-regulation.

Energy metabolism networks, including mitochondrial dysfunction and oxidative phosphorylation, had 55 and 42 dysregulated genes, respectively (Table S4). These include proteins of complex I (NADH, NDUF), complex II (SDHB), complex III (UQCRC1, UQCRH), complex IV (various isoforms of COX) and complex V (F_1_F_O_-ATP synthase) (Fig. 3A,B Table S4 and Fig. S2), mitochondrial dysfunction proteins (Fig. 3C), as well some other proteins associated with mitochondrial transport, such as TOMM22, TOMM5, TOMM6 and TOMM7 (Fig. 3C). In addition, we identified several upregulated genes involved in glycolysis (ENO1, FBP1, G6PD, GAPDH, ODC1, PDHB, PGD, PGK1, SDHB, TALDO1, TKT, TPI1, PFKL and ALDOA) as well as AKT1 and MYC, which have been reported as glucose metabolism regulators^18^ (Fig. 3C right panel).

This suggests that a mitochondrial/oxidative phosphorylation gene expression cassette is over-activated in the early stages of MTS.

### MCF-7 early-stage MTS exhibit a hyperactivation of oxidative mitochondrial metabolism

To corroborate the changes we found in the oxidative phosphorylation system (OxPhos), we measured the rate of oxygen consumption and the OxPhos flux. Cells from early-stage MCF-7 MTS exhibited a two-fold increase in basal respiration with respect to monolayer cells (Table 1). When OxPhos flux was unveiled by adding oligomycin (a specific F_1_F_O_-ATP synthase inhibitor), we observed that MCF-7 MTS had a 2-fold increase in ATP synthesis-coupled respiration with respect to monolayer cells (Table 1). In addition, adding the protonophore CCCP to the reaction chamber induced maximal uncoupled respiratory activity. This molecule dissipates the mitochondrial electrochemical protongradient (∆μ_H_^+^) and stimulates respiration to its maximum value. Once again, MTS exhibited a higher increase of oxygen consumption than monolayer cells, 17.2 ± 1.6 vs. 7.1 ± 1.1 ngAO·min^−1^·(1 × 10^6^ cells)^−1^, respectively (Table 1). A good parameter to estimate mitochondrial coupling is the ratio between the uncoupled respiration and basal respiration (U/B.R.). U/B.R. respiration values for MCF-7 MTS and monolayer cells were ~1.7 and ~1.3, respectively (Table 1). All of these results suggest that the mitochondria of MTS have higher OxPhos activity, i.e., a higher ATP synthesis coupled to higher respiratory chain activity (hyperactivation of oxidative mitochondrial metabolism).

### Changes in the cell cycle

**Figure 4:**
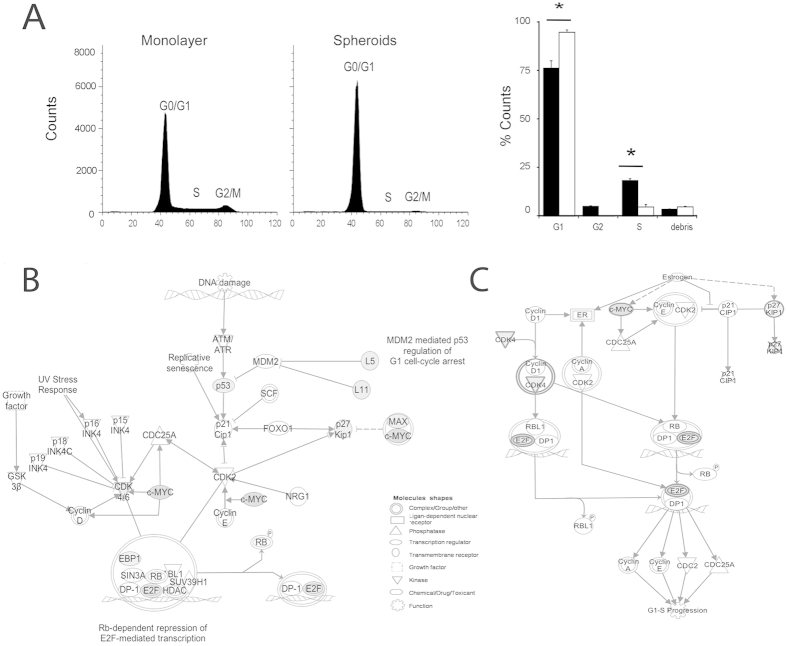
Cell-cycle measured in MTS using flow cytometry. (**A**) Analysis of the cell-cycle. *p < 0.05 n = 3 *t*-test. (**B**,**C**) Canonical pathways; G1/S checkpoint regulation and estrogen-mediated S phase. Entry (IPA). Interaction 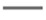 Direct, 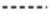 Indirect, 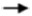 Modulation, 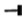 Inhibiting or/and ubiquitination, 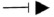 Inhibits and acts on, 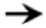 Reaction Gray colors up-regulation and intensity indicates the magnitude of change.

We also found enrichment of different pathways that affect the cell cycle. To validate this, we analyzed the cell cycle using flow cytometry and found that MTS had a substantial decrease in the number of cells found in the synthesis (S) and the Gap2 (G2) phases, with a concomitant increase in the number of cells in the Gap1 (G1) phase (Fig. 4A). The candidate molecules that could be regulating this phenotype in our experimental model were Tp53 Change to TP53, cMYC, p27KIP1 (CDKN1B), MAX/cMyc and CDK4, which were also involved in cell cycle G1/S checkpoint regulation (Fig. 4B, Table S5 and Fig. S3A) and in estrogen-mediated S-phase entry pathways (Fig. 4C, Table S5 and S3B). See Supplementary Table S5 and Fig. S3 for additional pathways.

### Long non-coding RNAs

**Figure 5:**
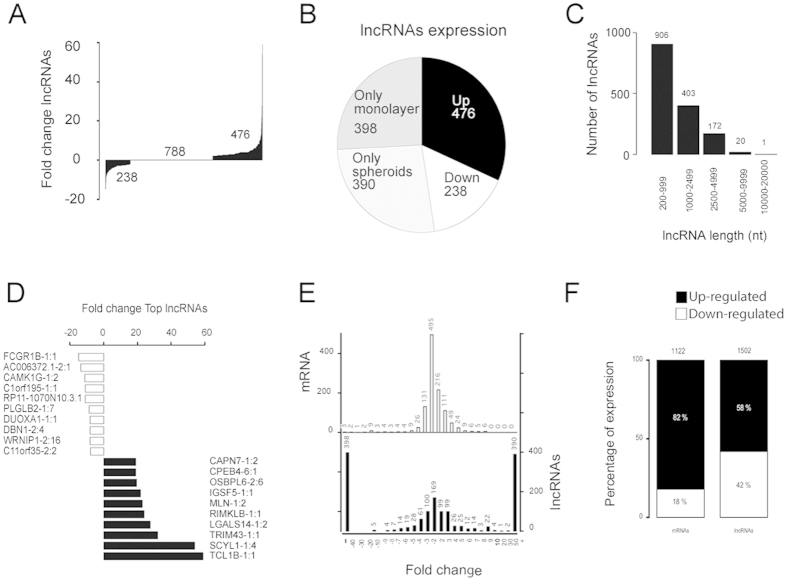
Analysis of the lncRNAs in MTS using RNAseq. (**A**) Fold change of all lncRNAs dysregulated. (**B**) A pie diagram of lncRNAs dysregulated included lncRNAs expressed in only one culture condition. (**C**) Length distribution of lncRNAs dysregulated. (**D**) Top ten dysregulated lncRNAs. (**E**) Histogram of the fold change distribution between dysregulated RNAs (**F**) Comparison of the lncRNAs and mRNAs dysregulated.

We then analyzed the expression of lncRNA transcripts in early-stage MTS. Fig. 5A,B show that 1502 lncRNAs were regulated, a very similar number to the total mRNAs altered (1122) (Fig. 5F). Eight-hundred and sixty-six lncRNAs (58%) were regulated positively (Fig. 5B,F), of which 390 were synthesized *de* novo in the MTS, as assessed with our methodology (Fig. 5B). Six-hundred and thirty-six lncRNAs (42%) were regulated negatively, of which 398 ceased to be expressed completely during the 6 days of MTS culture (Fig. 5F,B). We found a higher percentage of coding transcripts that were upregulated 82% (versus 58%) and downregulated 18% (versus 42%) (Fig. 5F).

In addition to this regulation, lncRNAs exhibited greater expression fold changes (up to 50-fold), in contrast to mRNAs (up to 5 fold), and nearly half of these lncRNAs were regulated in a binary manner (switched off completely or expressed *de novo*) (Fig. 5E).

Sixty-percent of the regulated lncRNAs (906) had a length of less than 1000 nt, 27% (403) were between 1000 to 2499 nt, and only 12% (172) were larger than 2500 nt (Fig. 5C). Notably, no differences were found in the strand transcribed for the regulated lncRNAs, 767 lncRNAs originated from the positive strand versus 735 from the negative strand, which was similar to all the lncRNAs (58271 positive strand and 53339 negative strand). The Chi-square test was equal to 0.0011 degrees of freedom and a p-value = 0.98172. In Fig. 5D, we present the top 10 positive and negative regulated lncRNAs in the MTS. lnc-TCL1B-1:1 was the most up regulated molecule, with a fold change of 59, whereas the most down regulated lncRNA was lnc-FCGR1B-1:1 with a fold change of −15.

**Figure 6:**
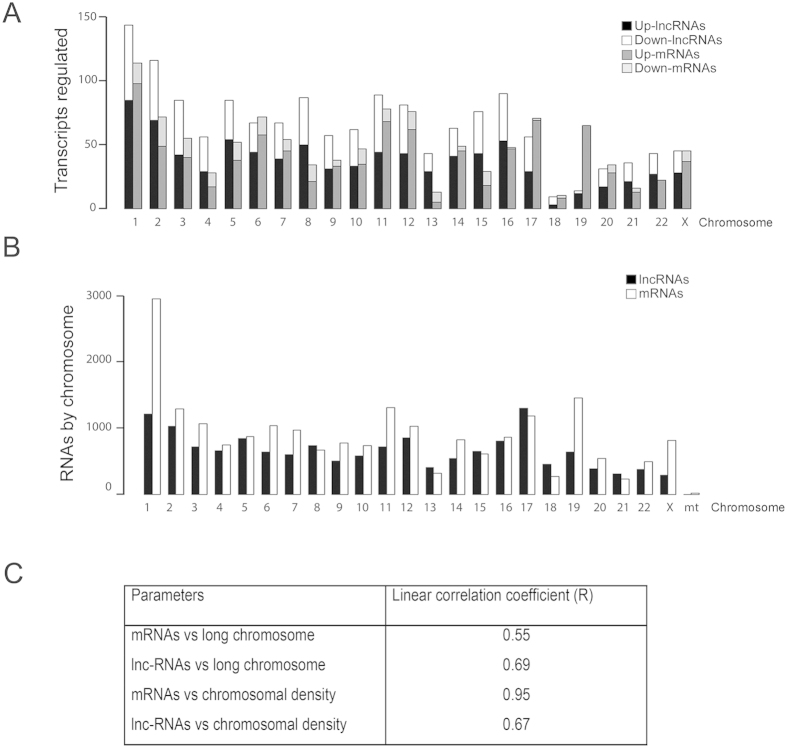
Distribution of lncRNAs and mRNAs differentially expressed by chromosome. (**A**) Up- and down- regulated mRNAs and lncRNAs dysregulated in MTS by chromosome. (**B**) Total lncRNAs and mRNAs by chromosome: Ensembl (http://www.emsembl.org/index.html). (**C**) Linear correlation coefficient (R) of mRNAs and lncRNAs according to length or chromosomal density.

With these data, we then sought to determine whether there were differences in the chromosomal localization of the regulated lncRNAs and mRNAs. In Fig. 6A, we show the distribution of dysregulated transcripts. No correlation was found between the number of mRNAs dysregulated and the length of each chromosome (R = 0.55), but, as expected, we found a good correlation between the numbers of regulated mRNAs versus the number of all described mRNAs present per chromosome (density) (R = 0.95) (Fig. 6C). Similarly, for lncRNAs, we also did not find a correlation between the number of regulated lncRNAs MTS along each chromosome (R = 0.69) (Fig. 6C), or their density per chromosome (R = 0.67) (Fig. 6C). In addition, we did not find a correlation between the number of regulated mRNAs and lncRNAs. Although both groups of RNAs are transcribed by RNA polymerase II, and the number of coding RNAs is very similar to the number of lncRNAs in each chromosome (Fig. 6B), we found that the number of lncRNAs and mRNAs regulated per chromosome were rather different in MTS, indicating a different regulation process for each group of transcripts (Fig. 6A).

**Figure 7:**
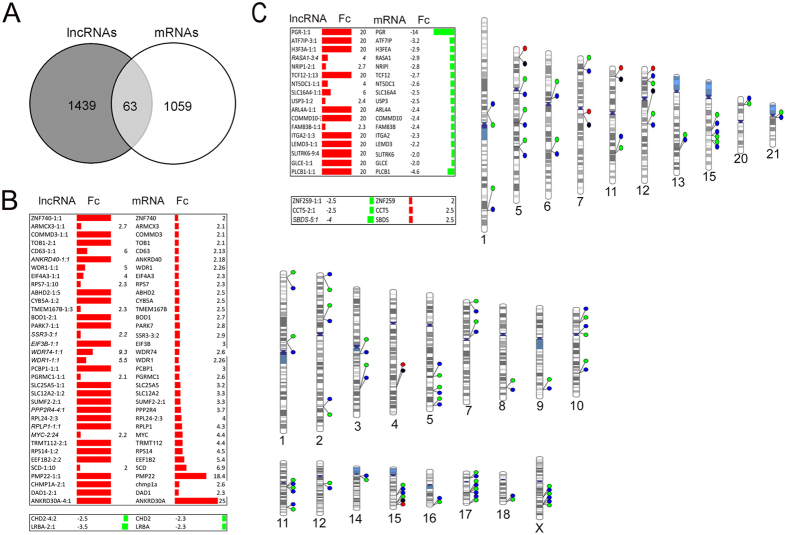
Relationship between dysregulated lncRNAs and neighbor mRNAs in MTS. (**A**) Venn diagram of 63 matching transcripts between mRNAs and lncRNAs according to their name in the LNCipedia database (isoforms of lncRNAs not included) (**B**) lncRNAs and neighboring mRNAs regulated in the same direction and phenogram. (**C**) lncRNAs and neighboring mRNAs genes regulated in opposite direction and phenogram. Fc = Fold change.

**Figure 8:**
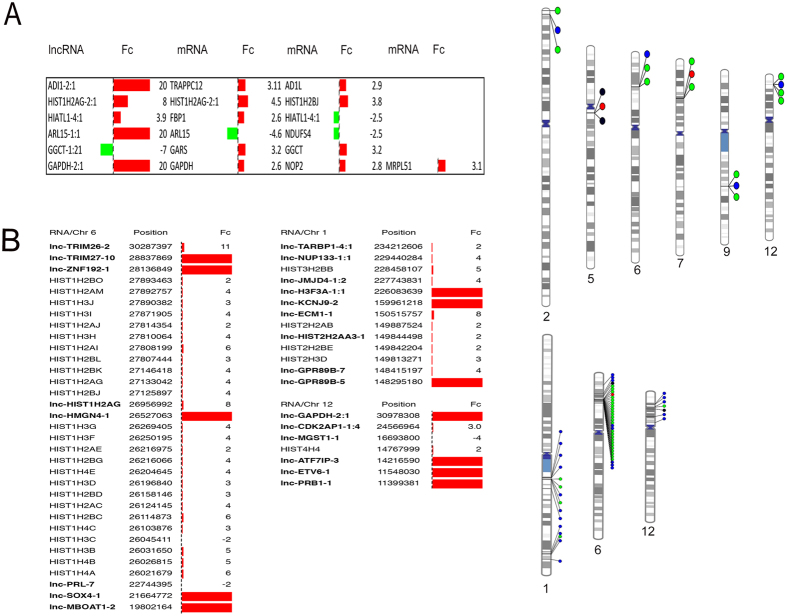
Relationship between dysregulated lncRNAs and neighbors mRNAs in MTS as assessed using the Integrative Genomics Viewer (IGV, Broad Institute). (**A**) lncRNAs with more than one neighbor mRNA regulated and the corresponding phenogram. (**B**) lncRNAs neighboring to histone cluster regulated in spheroid and the phenogram. Fc = Fold change.

Although lncRNAs are able to regulate genes located thousands of base pairs away, most of them carry out their function in nearby genes^19^. For this reason, we localized the nearest coding genes to the regulated lncRNAs and looked for co-regulation. Supplementary Table S6 shows the fold change and localization of 63 co-regulated lncRNAs and mRNAs in MTS cultivated for six days (Fig. 7A). We found 35 positively dysregulated lncRNA whose nearby genes were also increased (Fig. 7B). One of the most interesting was PMP22, which has been associated with breast cancer metastasis^20^. We found two lncRNAs with a concordant lower expression of their mRNA neighbors, CHD2 and LRBA (Fig. 7B). We also found 20 lncRNAs with an inverse expression pattern (Fig. 7C). Interestingly, 5 of these co-regulated lncRNAs were dysregulated with two neighboring genes that were also dysregulated (lncRNA-ADI1-2:1, lncRNA-HIST1H2AG-2:1, lncRNA-HIATL1-4:1, lncRNA-ARL15-1:1 and lncRNA-GGCT-1:21), and lncRNA-GAPDH with three neighboring dysregulated genes (GAPDH, NOP2 and MRPL51), indicating that this effect of lncRNA is possibly widespread (Fig. 8A).

We then sought to identify clusters of co-regulated genes in our data. As shown in Fig. 8B, we found three clusters located on chromosome 1, 6 and 12. These included two very interesting regulated histone clusters. The first was in chromosome 1, in which 4 modulated histone mRNAs are surrounded by several lncRNAs, and the second was in chromosome 6, with 25 histone genes, including HISTH2, HISTH3 and HISTH4, surrounded by several dysregulated lncRNAs (lnc-ZNF192-1:1, lnc-HIST1H2AG-2:1, lnc-HMGN4-1:1 and lnc-PRL-7:1) (Fig. 8B).

### Correlation between RNAm/lncRNA networks

It has been described that lncRNAs are able to regulate adjacent genes; using this approach, we found nodes for 63 lncRNA-mRNA co-regulated we found 63 lncRNA-mRNA co-regulated nodes (instead of we found nodes for 63 lncRNA-mRNA co-regulated) (previously mentioned). The main regulated biological network enriched in these nodes was the mTOR-signaling, MYC and HIF-1 cascade (Fig. S5).

Since its relative recent discover, there are only a handful described functions for the vast number of lncRNAs (Table S7), and even a lower characterized molecular targets for them. Nevertheless, we sought to analyze if there was information of at least some of the found deregulated lncRNAs. We found that 14 lncRNAs with established target genes in our model. These data were used to construct an interaction network using the lncRInter-lncRNA Interation and IPA tools. Interestingly, we found that 5 of the lincRNAs (LinC00467, MCM3AP-AST, PVT1, H19, and ZFas) converge at a MYC node and 6 more (PVT1, MALAT, GAS5, TPTG1, MEG3 y TUG1) converge at a p53 node, as shown in Fig. S6.

### Validation of mRNAs and lncRNAs

**Figure 9:**
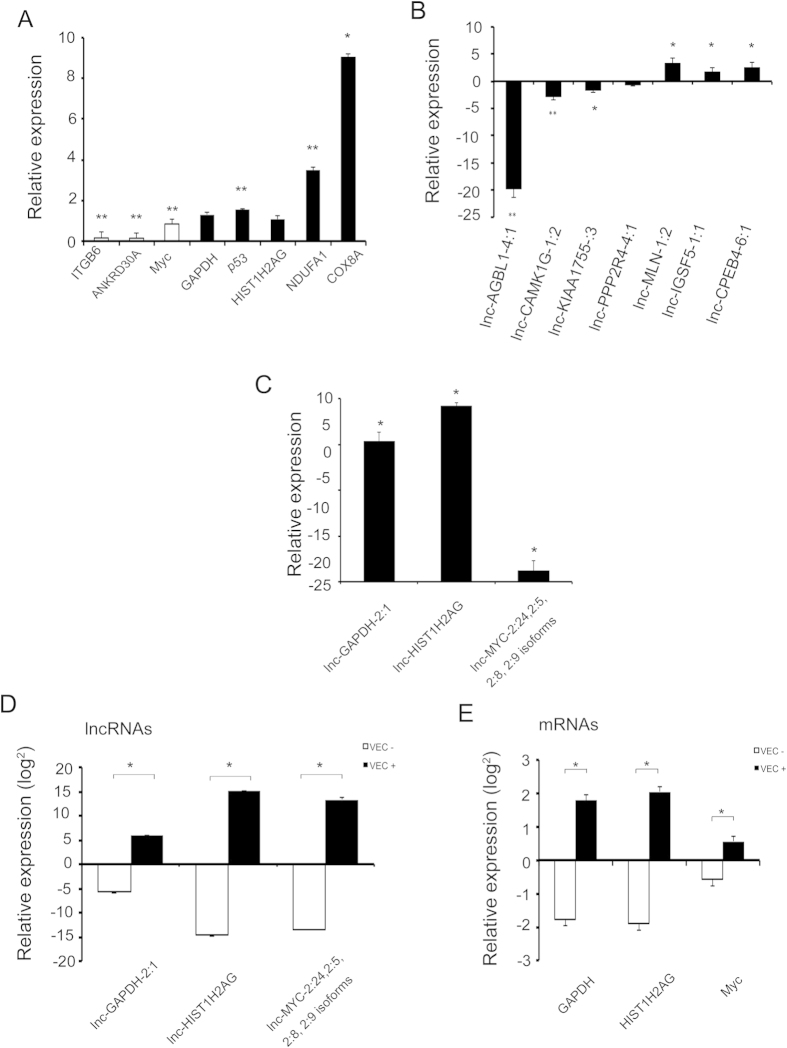
qPCR validation of lncRNAs and mRNAs of sequencing data and effect on mRNAs after of the lncRNAs overexpression. (**A**,**B**) qPCR validation of mRNAs and lncRNAs respectively. (**C**) qPCR validation of lncRNAs for cloning assays. (**D**,**E**) Level of lncRNAs and mRNAs following transfection with lncRNAs (VEC+) or empty vector (VEC−) for qPCR. *p < 0.05 and **p < 0.001.

When we validated 8mRNAs and 10 lncRNAs using qPCR, find success rate of 75 % for RNAs and 80 % for lncRNAs (Fig. 9B and C respectively). The values obtained by qPCR are shown in supplementary table S8 (Fig. 9A–C). And to To (instead of And to) assess the importance of these expressional changes, four pairs of lncRNAs/mRNAs pairs (lnc-GAPDH-2:1/GAPDH, lnc-HIST1H2AG-2:1/HIST1H2AG, lnc-MYC-2:24,2:5/MYC and lncANKDR30A/ANKDR30A) were validated by qRT-PCR (Fig. 9C). We were able to validate the co-regulation of three pairs, and failed to establish a co-regulation for the lncANKDR30A/ANKDR30A pair due to a large biological variability. To further support these results, we transiently overexpressed these four lncRNAs. Interestingly, we were able to reproduce the co-regulation in three lncRNA/mRNA pairs, which not only supports a possible new cis-acting function for these lncRNAs, but also validates our co-regulation approach for determining new lncRNAs targets (Fig. 9D,E and Table S9).

## Discussion

MTS represent an excellent model to study cancer biology because they mimic the behavior of tumors *in vivo*. Many of the phenotypic characteristics of cells grown in MTS are a consequence of gradients of nutrients and oxygen, which produces zonal heterogeneity and, in mature MTS, several cellular layers, an outer layer with proliferative cells, an intermediate layer containing quiescent cells and a central necrotic/apoptotic area. Nevertheless, avascular primary tumors and micro-metastasis are more accurately modeled by early stage MTS, in which the poor-hostile microenvironment of oxygen and nutrients are not properly established and most of the phenotype is derived by the presence of 3D cellular and extracellular matrix interactions.

To gain greater insight into the molecular mechanisms that are regulated during these early stages, we studied MTS with a diameter of approximately 200 microns in which there is no HIF1α expression, indicating the absence of hypoxia. To further validate our model, we analyzed the expression of p27Kip1, a protein that blocks the cell cycle at the G1/S phase checkpoint and is highly expressed in cells arrested at the G0 and G1 phases.p27Kip1 expression was limited to sparse cells, without forming a clear middle layer in the MTS, as would be expected in a hypoxic layer that is rich in quiescent cells of mature MTS . In our model, increased p27Kip1 expression in sparse cells could be induced by cell–cell contact^21^. In addition to this, we measured Ki67, a nuclear protein that is present during all active phases of the cell cycle (G1, S, G2 and mitosis), but absent from resting cells (G0). As expected, Ki67 positive cells were mainly located in the external layer.

We then sought to gain insight into the molecular mechanisms that could be regulated during this stage, and correlate this to the putative avascular tumor phenotype. Using massive RNA sequencing analysis we found 1122 dysregulated messenger RNAs in MTS of 6 days. As expected, among the top 20 regulated genes, all them were associated with cancer and eleven of them have been associated with breast cancer metastasis (i.e., NAALADL2, PMP22, CLDN4 and PGR) (Table S3)^20^,^22^,^23^,^24^.

These results strongly support the relevance of our model. Because we predicted that the main drivers for the phenotype would be related to cell-cell interactions, instead of hypoxic and/or cell cycle alterations, we focused on the regulation of membrane proteins. Indeed, we found that claudins, plexin 2, several integrins, syndecans, EPCAM and E –cadherin were regulated in the MTS. E-cadherin the major cadherin implicated in epithelial cell-cell adhesion, is associated with invasion and, more interestingly, it is commonly re-expressed by metastatic cancer cells to recover the adhesion complexes that are temporarily lost during the Epithelial-Mesenchymal Transition (EMT), which is required for effective dissemination^25^. Additionally, claudins are the major component of the tight junctions and are very important for the growth of metastatic cancer cells. For example, Abd-Elazeem M.A. and Abd-Elazeem M.A.^26^ reported high levels of claudin 4 expression in 66.1% of triple-negative breast cancer, particularly in invasive carcinoma, which correlated with the expression of Ki67. Similarly, Jiwa LS, *et al.*^23^, reported that Claudin-4 is overexpressed at high levels in breast cancer metastases and is thereby an attractive membrane bound molecular imaging and drug target^23^,^26^.

EPCAM is a transmembrane glycoprotein that plays an important role in carcinogenesis in many tumor types, including breast carcinoma, where high EPCAM expression is associated with larger tumors, nodal metastasis and worse survival^27^,^28^. Syndecans are members of the syndecan-proteoglycan family involved in cell binding and signaling and cytoskeletal organization. Syndecan-1 plays a vital role in regulating essential physiologic cellular functions, including proliferation and differentiation. This molecule is also involved in oncogenesis and metastatic dissemination of various malignant epithelial neoplasms, including breast cancer^29^,^30^. The changes in adhesion molecules could be playing a central role in the early stages of avascular tumors and micro-metastasis, as in our model.observed that the disaggregated spheroid cells showed a pattern of migration.

Previous research has extensively shown that cancer cells produce energy predominantly through a high rate of glycolysis, followed by cytosolic lactic acid production, as opposed to oxidation of pyruvate in mitochondria, a phenomenon termed the Warburg Effect^31^. Interestingly, we found a paradoxical respiratory increase in our model. Two pathways were associated with overactive oxidative phosphorylation respiration in mitochondria and mitochondrial dysfunction that included 55 mitochondrial genes (Table S4). We corroborated this result by measuring mitochondrial respiration and found that it increased two-fold in MTS. This hyperactivation of oxidative mitochondrial metabolism has been described in microenvironment-regulated tumor metabolism, where tumor stroma and adjacent host tissues are catabolic, and as a consequence, cancer cells are anabolic^32^,^33^,^34^. In this model, energy is transferred from the catabolic compartment to the anabolic compartment via sharing of nutrients (onco-metabolites) that promote tumor growth. The two-compartment tumor metabolism model has been demonstrated in fibroblasts and breast cancer cells, and more recently between adipocytes and ovarian cancer cells^35^.This effect can be phenocopied by incubating cancer cells alone with L-lactate^34^.

This is the first report that fully analyzed the transcriptome of early (6 days) MTS stages. Among the results, we found that 55 dysregulated genes were associated with mitochondria. Rodríguez-Enríquez S *et al.*, 2008 reported that in intermediate-stage MTS (11 day) the ATP supply was provided by both energy pathways (OxPhos and glycolysis) and, at this stage, the OxPhos flux was similar to that observed in cells cultured as monolayers and it decreased in older spheroids (15 days) in which the glycolysis increased significantly, reproducing the Warburg effect^36^. The present results, showing an increase of oxidative phosphorylation at very early stages and deregulation of several metastatic genes and increased cellular migration suggest that both processes are linked. The use of early stage spheroids would be a good model for initial metastatic seeding.

Energy production is strongly associated with tumor cell processes such as apoptosis, metastasis and chemo resistance. The activation of OxPhos complexes provides an opportunity to use specific drugs directed toward these complexes for an antineoplastic effect. For example, metformin behaves as a weak mitochondrial poison because of its Complex I inhibitor activity, and metformin has been evaluated as an anti-cancer agent in preclinical animal models. Similarly, both malonate, which inhibits Complex II and azide, which is an inhibitor of Complex IV could be further developed for clinical testing. In the present report we also found a deregulation of genes associated with glycolysis, although to a lesser extent to OxPhos genes. It will be of great interest to analyze the relative participation of each process in the full energy metabolism of early-stage MTS, since this could have clinical implications.

Because the small size of the MTS precludes the presence of hypoxia, we hypothesize that these changes are mainly based on changes in the number and types of cell-cell interactions and the microenvironment. This latter change would be the result of the presence of new metabolites and/or pH changes that are being generated. Further experiments are needed to test the relative contribution of each phenomenon.

We also sought to investigate whether the three-dimensional structure could be affecting the expression of lncRNAs. Our analyses found a similar number of dysregulated lncRNAs and mRNAs. Many of these were synthetized *de novo* or downregulated to non-detectable levels. This binary behavior contrasted with mRNA expression patterns in which a wide range of expression changes was found. In addition, lncRNAs are less abundant than coding RNAs. Arjun Raj *et al.*, 2015, when analyzing the distribution of single cell counts, demonstrated the relatively low overall expression of lncRNAs, with 43% of lncRNA-cell pairs having 10 or fewer molecules per cell on average and with a median of 14 molecules across all gene-cell-pair distribution medians (vs 36 for the 49 mRNA-cell pairs we examined)^37^. This finding could account for the binary behavior of lncRNAs.

Previous research has shown that although lncRNAs are able to regulate genes located thousands of base pairs away, most of them carry out their function in nearby genes^19^. For this reason, we located the nearest coding genes to the regulated lncRNAs and looked for co-regulation. We found 63 co-regulated lncRNAs and mRNAs. Of these, 35 were positively co-regulated, and one is particularly of interest. PMP22 is a messenger RNA that has been associated with breast cancer and metastasis^20^. Twenty lncRNAs had a change in the opposite direction with regards to a neighboring gene (Fig. 7C), and six lncRNAs (Fig. 8A) were co-regulated with two or three neighbor genes. Among these, lnc-GAPDH was co-regulated with three neighbor genes (GAPDH, MRPL51 and NOP2). GAPDH is an enzyme that, in addition to catalyzing the sixth step of glycolysis in the cytoplasm, has been shown to be involved in many cellular processes, including DNA repair, tRNA export, membrane fusion and transport, cytoskeletal dynamics and cell death. Interestingly, GAPDH has been found in particulate fractions of mitochondria with low levels that are rapidly increased under stressed conditions, such as serum deprivation and exposure to DNA-damaging agents^38^,^39^. Mitochondrial GAPDH interacts with voltage-dependent anion channel 1 and regulates mitochondrial membrane potential and cell survival^40^. Previous research has demonstrated that rotenone, a common mitochondrial complex I inhibitor, induces GAPDH enrichment in particulate fractions^40^. Further work is needed to assess whether lnc-GAPDH, which was one of the most dysregulated lncRNAs, could be concomitantly regulating the expression of all or at least some of its neighbor dysregulated genes.

Of particular interest, due to finding hyperactivation of OxPhos, is the coregulation of several lncRNAs and neighboring genes that are associated with mitochondrial function, including Gamma-glutamylcyclotransferase (GGCT)^41^, Shwachman-Bodian-Diamond syndrome SBDS^42^, Parkinson protein 7, PARK7^43^, progesterone receptor PGR^44^, Cytochrome b type B (CYB5B)^45^, ribosomal protein S14^46^, ADP-ribosylation factor/ARF-like protein 4 (ARF4/ARL4)^47^ and Solute Carrier Family 12 (Sodium/Potassium/Chloride Transporter), Member 2^48^. Finally, it is worth mentioning that we identified three co-regulated lnc-messenger RNA histone clusters on chromosomes 1, 6 and 12. These lncRNAs could be operating as enhancer-lncRNAs. Further molecular work is needed to evaluate this idea.

Finally the analysis of the interaction between the two networks mRNA and lncRNA we found that 14 lncRNAs which their molecular targets are known, 5 of them converge in MYC network and 6 converge in p53 network.

In summary, in this study we describe the coding and non-coding transcriptome of early breast cancer MTS three main groups of genes were dysregulated in this experimental model. The first set involved membrane proteins implicated in cell-cell junctions, such as claudins, plexin2, integrins, syndecans, EPCAM and E-cadherin. In addition to a migration pattern in cells spheroids. The second set involved metastasis genes, and the third set consisted of genes belonging to mitochondrial complexes. The regulation of these genes was associated with hyperactivation of OxPhos, which was confirmed in functional assays. In addition to coding RNAs, we found a clear deregulation of several lncRNAs, mostly in a binary mode. Several lncRNAs were co-regulated with neighboring genes, pointing toward new avenues for research. In particular histone coding and non-coding RNAs on three chromosomes were co-regulated, which is of interest to explore further.

## Materials and Methods

### Cell line culture

Breast cancer cells (MCF-7) obtained from the American Type Culture Collection # HTB-22 (ATCC, Manassa, VA, USA) were cultured in EMEM (Eagle’s Minimum Essential Medium) containing 2 mM L-glutamine and 1 mM sodium pyruvate (ATCC #30-2003), supplemented with 5% fetal bovine serum (ATCC #30-2020), 100 I.U./ml penicillin and 5 μg/ml streptomycin (ATCC #30-2300), at 37 °C in 5% CO_2_.

### Generation of MTS

MT formation of the MCF-7 cell line was performed using the liquid overlay technique with some modifications^49^. Briefly, 1 × 10^6^ cells were seeded in 12.5 cm^2^ culture flasks (#sc-200257, UltraCruz, Biotechnology, CA, USA,) adjusting to a final volume of 4 ml with Leibovitz’s L-15 medium (ATCC #30-2008) supplemented with 5% fetal bovine serum. The flasks were placed in an orbital incubator at 37 °C with constant agitation at 54 rpm for six days, renewing the medium at 24 h and then every 48 h. The size of the MTS was determined by collecting a sample at two, four, six and eight days of culture. They were then fixed with 4% formaldehyde in phosphate buffered saline (PBS). Following this, the two orthogonal diameters of 30 MTS were measured daily, using an inverted AXIO Scope A1 and Axiovision version 4.8.2.0 software (Carl Zeiss, Jena, Germany) microscope. The mean geometric diameter was determined based on the formula D_G_ where D_G_ = √a × b^49^. Individual assay was performed in triplicate.

### Histology of MTS

Fixed MTS where embedded in paraffin and sectioned (5 μm). For the characterization of the MTS, the sections were processed and evaluated using different techniques. Staining was performed using 4′, 6-diamidino-2-phenylindole (DAPI) or Mayer’s hematoxylin (H&E) to visualize nuclei or the general structure of the MTS. Parallel incubation with primary antibodies diluted in bovine serum albumin was conducted using a 1:200 dilution for anti-Ki67 and 1:100 for anti-p27Kip1 and anti-HIF 1-α. Antibody binding was then visualized using the standard avidin-biotin-peroxidase complex technique (#K0690 LSAB™+ Kits, Universal Dako, Sao Paulo, Brazil) counterstained with Mayer’s hematoxylin. The antibodies used were, Anti-HIF 1-α (Hypoxia-inducible factor-1, AB3883 Upstate Millipore), anti-p27Kip1 (cyclin-dependent kinase inhibitor 1B, ab54563 abcam) and anti-Ki67 (marker of proliferation Ki-67, AB9260 Millipore Chemicon, Jaffrey, USA).

### Migration assays

Cells grown as a monolayer and 6 days MTS were disaggregated with Accumax (Chemicon, MA, USA. #SCR006) were seeded and subjected to real-time migration monitoring using the CIM-Plate 16 and xCELLigence System using a RTCA DP Instrument (ACEA Biosciences, Inc, San Diego CA, 92121 USA). In this system, 40 000 cells of each culture condition were seeded in the upper chamber of a CIM-Plate 16 without FBS. The upper chamber was then placed on the lower chamber of the CIM-Plate 16 containing growth medium supplemented with 10% FBS as an attractant, medium without FBS (negative control). Cell migration was monitored over a period of 24 to 30 hours. MDA-MB-231 cells were used as a positive control (Fig. S1). Three assays were performed for each culture condition. Statistical analysis was performed using *t*-tests.

### Cell cycle analysis

Monolayer cells and MTS after six days of culture were dispersed using Accumax (#SCR006 Chemicon) in EMEM medium with 5% fetal bovine serum and filtered through a 70 μm mesh (# 352350, Falcon Corning, Tewksbury, MA, USA). The cell cycle was determined by flow cytometry using the DNA Reagent Kit (Cycletest Plus #340242, BD, Billerica, MA) following the manufacturer’s recommendations. The samples were processed in a BD FACSAria Cell Sorter 3 and analyzed using the ModFit LT 3.2 version software. Each individual assay was performed in triplicate. Statistical comparisons were performed using *t*-tests.

### Purification of total RNA from MCF-7 cells

Total RNA was isolated from monolayer and MTS cells cultured for six days using the PARIS Kit (#AM1921, Applied Biosystems, Woburn, MA, USA) following the manufacturer’s recommendations, and excluding RNA less than 200 bp. RNA concentration and quality was determined by a combination of spectrophotometry (NanoDrop 1000 Spectrophotometer, Thermo Scientific, Tewksbury, MA, USA), and automated capillary electrophoresis (Bioanalyzer 2100, Agilent Technologies, Inc., Santa Clara, CA) using the Agilent RNA 6000 Nano. RIN values obtained were higher than 9.0 in all cases. Ribosomal RNA was removed by means of RiboMinus Eukaryote Kit (#A1083708, Invitrogen, Oregon, USA,) following manufacturer´s instructions. Effective ribosomal RNA removal was corroborated using capillary electrophoresis.

### RNA transcriptome

We constructed libraries with RNA isolated from MTS and monolayer cells at six days in culture using TruSeq RNA sample preparation (RS-122-2001, Illumina, San Diego, CA, USA) following the recommendations of the manufacturer, quantified by fluorometry (Qubit fluorometer, Invitrogen) and sequenced in a GAIIx machine (Illumina). We performed 2 × 72 bp runs. We obtained a total of 30,467,063 reads for the monolayer cultures and 24,363,843 reads for MTS cultures. After cleaning the reads, the RNA-Seq data were analyzed using the CLC genomics workbench (version 7 CLC Bio Cambridge, MA). The following parameters were used: alignment with 2 maximum number of mismatches; a 0.9 minimum length fraction; a 0.8 minimum similarity fraction; and 10 maximum hits for a read. Mapped reads range was 16 × 106–28 × 106. All transcripts with a relative expression level lower than 0.2 and less than 10 reads were filtered out. Empirical analysis of differential gene expression was calculated using EdgeR algorithm^50^.

### qPCR validation of lncRNAs

We randomly selected three up-regulated and four down-regulated lncRNAs with >2.0-fold change for validation. In addition, 8 mRNAs associated to mitochondria or cell cycle were arbitrarily selected and 4 lncRNAs/mRNAs co-regulated to additional validatation. The nucleotide sequence of each lncRNA was obtained from LNCipedia 3.0 (http://www.lncipedia.org/) and the following specific primers for PCR were designed using Primer Express 3.0 software (Applied Biosystems, Carlsbad, CA) (see Table S1) cDNA was synthesized from 2 μg total RNA using the Maxima First Strand cDNA Synthesis Kit for RT-qPCR (#K1642 Thermo Scientific) and a temperature of 65 °C, as recommended for RNAs that have a large amount of secondary structure. A semiquantitative PCR was first performed to standardize the amplification and to provide enough DNA for subsequent steps. The correct amplification for all the amplicons was then verified using Sanger capillary Applied Biosystems 3730xl DNA Analyzer. Then, qPCR was performed using the SYBR Select Master Mix Maxima (#4472908 Applied Biosystems). Analysis was carried out using a 7500 Real-Time PCR Systems and the sequence detector 1.4 version software (Applied Biosystems). The expression level of each lncRNA was represented as relative expression, using relative standard curve methods. For mRNAs, the relative gene expression was calculated by the standard ∆∆Ct method. Differential expression between monolayers and MTS were analyzed using ANOVA for lncRNAs and *t-*tests for paired data for mRNAs. Six replicates were performed in each condition for lncRNA and three independent assays for mRNAs. A value of p < 0.05 was considered significant. We used *TBP* (TATA Box Binding Protein) as an endogenous control, because it is regulated less than GAPDH under different cellular conditions and exhibits low expression in MCF-7 cells^51^.

### Oxygen consumption and determination of OxPhos flux

To determine oxygen consumption, MCF-7 monolayer culture (80% confluence) cells or MTS cells from 6 days of culture were disrupted with 3 ml of accutase (#00-45555-56, eBioscience) for 5 minutes at 37 °C. Cells were collected, gently washed with fresh medium and centrifuged at 1,200 rpm for 5 minutes at 37 °C. Then, the cells were suspended in 200–300 μL of Krebs-Ringer medium (125 mM NaCl, 5 mM KCl, 1 mM MgCl2, 1 mM KH2PO4, 25 mM HEPES, 1.4 mM CaCl2, pH = 7.2). Rates of oxygen consumption were measured in a Strathkelvin Instruments 782 Oxygen Meter (North Lanarkshire, Scotland, UK) interfaced with a computer. Samples were placed in a water-jacketed chamber at 37 °C. Basal respiration rates were obtained by adding 1 × 10^6^ of either MCF-7 monolayer or MTS cells from 6 days of culture to 200 μl of air-saturated Krebs-Ringer medium. 5 μM oligomycin or 6 μM carbonyl cyanide m-chlorophenylhydrazone (CCCP) were added to determine oxidative phosphorylation (OxPhos) flux and full mitochondrial uncoupled respiration, respectively. In addition, 1 mM NaCN was added to fully inhibit mitochondrial oxygen consumption. Five assays were performed with each culture condition. Statistical analysis was performed using *t*-tests.

### Overexpression of lncRNAs

DNAs from lnc-MYC-2:5, lnc-GAPDH-2:1, lncHIST1H2AG-2:1 and lnc-ANKRD30A-4:1 were cloned in the pQCXIP vector. Their sequences were synthetized as gBlocks (Integrated DNA technologies, IA, USA), designed to share 15 bp of homology at their ends with the vector digested with the restriction enzyme EcoRI (New England Biolabs, MA, USA. R101S). Ligation of the vector-gblocks was carried out using the In-Fusion HD Cloning Plus CE kit (Clontech, CA, USA. 638916). The plasmids were transfected into MCF-7 cells using the Transfection reagent X-Fect kit (Clontech, CA, USA. 631317) according to manufacturer’s recommendations. Briefly, one day prior to transfection MCF7 cells were seeded at approximately 70% confluence in 6-well plates. Cells were transfected with 5 μg of each vector and 1.5 μL of the X-Fect Transfection Reagent. Forty-eight hours post-transfection, the RNA was extracted to evaluate the levels of each transcript.

## Additional Information

**How to cite this article**: Pacheco-Marín, R. *et al.* Transcriptome profile of the early stages of breast cancer tumoral spheroids. *Sci. Rep.* **6**, 23373; doi: 10.1038/srep23373 (2016).

## Supplementary Material

Supplementary Information

## Figures and Tables

**Table 1:** Rates of oxygen consumption and OxPhos flux in MCF-7 monolayer and 6-day MTS.

	Monolayer cells	6-day MTS
Basal respiration (B.R.)^a^	5.2 ± 2	10.3 ± 2.7***
+5 μM oligomycin	1.7 ± 0.4	3 ± 0.4**
+1 mM NaCN^b^	0.6 ± 0.1	0.6 ± 0.3
OxPhos flux^c^	3.5 ± 1.8	7.3 ± 2.5**
Uncoupled respiration (U)^d^	7.1 ± 1.1	17.2 ± 1.6**
U/B.R.	1.3 ± 0.1	1.7 ± 0.1*

All values are shown in ngAO/min/10^6^ cells. Data from five independent experiments are expressed as the mean ± SD. *t-* tests for paired data: *<0.05, **<0.005, ***<0.0005.

^a^Basal respiration was determined in the absence of exogenous electron donors or carbon sources.

^b^NaCN was added to full inhibit mitochondrial oxygen consumption.

^c^OxPhos flux = Basal respiration rate - rate of oxygen consumption in the presence of oligomycin.

^d^Uncoupled respiration (U) was induced with 6 μM CCCP.
