# Supplementary material for: Transcriptome profile of the early stages of breast cancer tumoral spheroids
Source: Sci Rep. 2016 Mar 29;6:23373. doi: 10.1038/srep23373 (PMC4810430; doi:10.1038/srep23373)
Supplement: Supplementary Information [file srep23373-s1.pdf]

Transcriptome profile of the early stages of breast cancer tumoral spheroids

Rosario Pacheco-Marín [a,e], Jorge Melendez-Zajgla [b], Gonzalo Castillo-Rojas [c], Edna Mandujano-Tinoco [b], Alfredo Garcia-Venzor [b], Salvador Uribe-Carvajal [d], Alfredo Cabrera-Orefice [d], Carolina Gonzalez-Torres [b], Javier Gaytan-Cervantes [b], Irma B Mitre-Aguilar [f] and Vilma Maldonado[a]*.

[a] Epigenetics and [b] Functional Genomics laboratories, National Institute of Genomic Medicine. Periférico Sur No.4809, Col Arenal Tepepan, Delegación Tlalpan, México, D.F., C.P 14610

[c] Microbial Molecular Immunology Program, Department of Microbiology and Parasitology. Faculty of Medicine, National Autonomous University of Mexico (UNAM), University City Avenue 3000 C.P. 04510, Coyoacan, Mexico City.

[d] Department of Molecular Genetics, Institute of Cellular Physiology (UNAM), University City Avenue 3000 C.P. 04510, Coyoacan, Mexico City.

[e] Posgraduate Program in Biological Sciences, Faculty of Medicine (UNAM), University City Avenue 3000 C.P. 04510, Coyoacan, Mexico City.

[f] Unit of Biochemistry, National Institute of Medical Sciences and Nutrition Salvador Zubirán (INCMNSZ), Av. Vasco de Quiroga Nº 15, Colonia Belisario Domínguez Sección XVI, Delegación Tlalpan. CP.14080, México D. F., México

* Correspondence to vilmaml@gmail.com

Supplementary Table S1. Primers of lncRNAs, mRNAs and endogenous control (TBP)

GEN	Sequence forward	Sequence reverse
lnc-CAMK1G-1:2	5´-CTCCCTGCTTCGGGTCAGA-3´	5´-GCCCCATAAAAGGCTGTTCTC-3´
lnc-PPP2R4-4:1	5´-CCCTGCACGTGGATATTAGAGTTT-3´	5´-GGGACCTTGCACCTACAAAATC-3´
lnc-AGBL1-4:1	5´-AATGGCAACCATCAAATCAAGA-3´	5´-GCAAAGCATCACAATTCATGGA-3´
lnc-KIAA1755-5:3	5´-TCTCGAAATCTGCAGACATCTTG-3´	5´-GTGAGAGTGGAGCCGACCAT-3´
lnc-IGSF5-1:1	5´-GGGCCACAACTCAACCCATA-3´	5´-GCTGGTCGTTGCTCAAGAAAC-3´
lnc-MLN-1:2	5´-TTCAGGCCCCGAGTTTCTC-3´	5´-GGGCAAGGGCAGAGTAGGA-3´
lnc-CPEB4-6:1	5´- ACCTCTCCTGCGTTTGATCTG-3´	5´-GGCCGTGGGCAAGTTACTT-3´
lnc-MYC-2:24, 2:5, 2:8 and 2:9 isoforms	5´-CTTCCAGTGGATTTCCTTGC-3´	5´-TTTCTCCCAGACACGGATTC-3´
lnc-GAPDH-2:1	5´-GGCTGTTGTGATTCAGTTGG-3´	5´-ATCGCCCACCAAGATTACAG-3´
lnc-HIST1H2AG-2:1	5´-ATGGTTTGCAGAGGCTCAAG-3´	5´-TGGGTGACAGGTTTGATGTC-3´
lnc-ANKRD30A-4:1	5´-TGCTGCAGGCAATTATGC-3´	5´-GCTTTTTCCAAAGGGGTTG-3´
MYC	5´-GTTTGCCATTACCGGTTCTC-3´	5´-TGCAGAAGGTCCGAAGAAAG-3´
GAPDH	5´-CAATGACCCCTTCATTGACC-3´	5´-ATGACAAGCTTCCCGTTCTC-3´
HIST1H2AG	5´-TGCCCAAAAAGACTGAGAGC-3´	5´-AAAGAGCCGTTGGTTTGGAC-3´
ANKRD30A	5´-AACACGGCTCTCCATTATGC-3´	5´-TAGCCTTGTTGTGCACTTCG-3´
COX8A	5´-TCCCCTGGATCATGTCATTC-3´	5´-CACCATGGAGACATCAAGAGG-3´
NDUFA1	5´-CGTGTGCTTGTTGATTCCAG-3´	5´-TGCGCCTATCTCTTTCCATC-3´
TP53	5´-GTCTTTGAACCCTTGCTTGC-3´	5´-CCACAACAAAACACCAGTGC-3´
ITGB6	5´-CGACTGTGACTGTGGTGAAT-3´	5´-CTTTGGTTCAGAAATGCAAG-3´
TBP	5´-CCACAGCTCTTCCACTCACA -3´	5´-CTCATGATTACCGCAGCAAA -3´

Supplementary Table S2. Cell index between monolayers and spheroids disaggregated, as well as MDA-MB- 231 monolayer cells (Positive control)

**Culture condition**	**Time**			
	**24h**		**30h**	
	Cell index	p-value	Cell index	p-value
Monolayer	0.02 ± 0.01	<0.0001	0.02 ± 0.01	<0.0001
Spheroids disaggregated 6 days	0.26 ± 0.11		0.42 ± 0.04	
MDA-MB-231	1.684 ± 0.11		1.797 ± 0.07	

Supplementary Table S3. Genes mRNAs top up/down related to breast cancer and metastasis

GEN	Name	References
**CLDN4**[*]	Claudin 4	[1-4]
FST[*]	Follistatin	[5-8]
**MIF**[*]	Macrophage migration inhibitory factor (glycosylation-inhibiting factor)	[9,10]
NPY1R[*]	Neuropeptide Y receptor Y1	[11]
PGR[*]	Progesterone receptor	[12]
**PMP22**[*]	Peripheral myelin protein 22	[13-15]
S100A9[*]	S100 calcium binding protein A9	[16]
**TFF1**[*]	Trefoil factor 1	[17]
RERG[*]	RAS-like, estrogen-regulated, growth inhibitor	[18]
COL12A1*	Collagen, type XII, alpha 1	[19]
**COL4A5***	Collagen, type IV, alpha 5	[20,21]
**S100A6**	S100 calcium binding protein A6	[22]
**RPS12**	Ribosomal protein S12	[23,24]
NAALADL2	N-acetylated alpha-linked acidic dipeptidase-like 2	[25]
**IFIT2**	Interferon-induced protein with tetratricopeptide repeats 2	[26,27]
**ATP5E**	ATP synthase, H+ transporting, mitochondrial F1 complex	[28]
ANKRD1	Ankyrin repeat domain 1 (cardiac muscle)	[29]
**RPS21**	Ribosomal Protein S21	[30]
POF1B	Premature ovarian failure, 1B	[31]
**RPL36AL**	Ribosomal protein L36a-like	[32]

[*] Related genes in breast cáncer metastasis and down-regulated are in bold letters. *See references on next page*

Reference

1 Jiwa, L. S. *et al.* Upregulation of Claudin-4, CAIX and GLUT-1 in distant breast cancer metastases. *BMC Cancer* **14**, 864 (2014).

2 Abd-Elazeem, M. A. & Abd-Elazeem, M. A. Claudin 4 expression in triple-negative breast cancer: correlation with androgen receptors and Ki-67 expression. *Ann Diagn Pathol* **19**, 37-42 (2015).

3 Lanigan, F. *et al.* Increased claudin-4 expression is associated with poor prognosis and high tumour grade in breast cancer. *Int J Cancer* **124**, 2088-2097 (2009).

4 Blanchard, A. A. *et al.* Differential expression of claudin 1, 3, and 4 during normal mammary gland development in the mouse. *DNA Cell Biol* **25**, 79-86 (2006).

5 Mock, K. *et al.* The EMT-activator ZEB1 induces bone metastasis associated genes including BMP-inhibitors. *Oncotarget* **6**, 14399-14412 (2015).

6 Ohta, N. *et al.* Human umbilical cord matrix mesenchymal stem cells suppress the growth of breast cancer by expression of tumor suppressor genes. *PLoS One* **10**, e0123756 (2015).

7 Sengupta, D. *et al.* ERRbeta signalling through FST and BCAS2 inhibits cellular proliferation in breast cancer cells. *Br J Cancer* **110**, 2144-2158 (2014).

8 Karagiannis, G. S., Berk, A., Dimitromanolakis, A. & Diamandis, E. P. Enrichment map profiling of the cancer invasion front suggests regulation of colorectal cancer progression by the bone morphogenetic protein antagonist, gremlin-1. *Mol Oncol* **7**, 826-839 (2013).

9 Verjans, E. *et al.* Dual role of macrophage migration inhibitory factor (MIF) in human breast cancer. *BMC Cancer* **9**, 230 (2009).

10 Zhang, M., Yan, L. & Kim, J. A. Modulating mammary tumor growth, metastasis and immunosuppression by siRNA-induced MIF reduction in tumor microenvironment. *Cancer Gene Ther* **22**, 463-474 (2015).

11 Liu, L. *et al.* NPY1R is a novel peripheral blood marker predictive of metastasis and prognosis in breast cancer patients. *Oncol Lett* **9**, 891-896 (2015).

12 Aurilio, G. *et al.* A meta-analysis of oestrogen receptor, progesterone receptor and human epidermal growth factor receptor 2 discordance between primary breast cancer and metastases. *Eur J Cancer* **50**, 277-289 (2014).

13 Mimori, K. *et al.* Identification of molecular markers for metastasis-related genes in primary breast cancer cells. *Clin Exp Metastasis* **22**, 59-67 (2005).

14 Winslow, S., Leandersson, K. & Larsson, C. Regulation of PMP22 mRNA by G3BP1 affects cell proliferation in breast cancer cells. *Mol Cancer* **12**, 156 (2013).

15 Tong, D. *et al.* Gene expression of PMP22 is an independent prognostic factor for disease-free and overall survival in breast cancer patients. *BMC Cancer* **10**, 682 (2010).

16 Gumireddy, K. *et al.* ID1 promotes breast cancer metastasis by S100A9 regulation. *Mol Cancer Res* **12**, 1334-1343 (2014).

17 Wang, H. *et al.* Gene expression markers in circulating tumor cells may predict bone metastasis and response to hormonal treatment in breast cancer. *Mol Clin Oncol* **1**, 1031-1038 (2013).

18 Habashy, H. O. *et al.* RERG (Ras-like, oestrogen-regulated, growth-inhibitor) expression in breast cancer: a marker of ER-positive luminal-like subtype. *Breast Cancer Res Treat* **128**, 315-326 (2011).
19 Dalgin, G. S. *et al.* Portraits of breast cancer progression. *BMC Bioinformatics* **8**, 291 (2007).
20 Nakano, S. *et al.* Differential tissular expression and localization of type IV collagen alpha1(IV), alpha2(IV), alpha5(IV), and alpha6(IV) chains and their mRNA in normal breast and in benign and malignant breast tumors. *Lab Invest* **79**, 281-292 (1999).
21 Wong, N. A., Wingate, J. & Colling, R. A study of alpha5 chain of collagen IV, caldesmon, placental alkaline phosphatase and smoothelin as immunohistochemical markers of gastrointestinal smooth muscle neoplasms. *J Clin Pathol* **67**, 105-111 (2014).
22 McKiernan, E., McDermott, E. W., Evoy, D., Crown, J. & Duffy, M. J. The role of S100 genes in breast cancer progression. *Tumour Biol* **32**, 441-450 (2011).
23 Deng, S. S. *et al.* Comparative proteome analysis of breast cancer and adjacent normal breast tissues in human. *Genomics Proteomics Bioinformatics* **4**, 165-172 (2006).
24 Chen, D. *et al.* RPS12-specific shRNA inhibits the proliferation, migration of BGC823 gastric cancer cells with S100A4 as a downstream effector. *Int J Oncol* **42**, 1763-1769 (2013).
25 Whitaker, H. C. *et al.* N-acetyl-L-aspartyl-L-glutamate peptidase-like 2 is overexpressed in cancer and promotes a pro-migratory and pro-metastatic phenotype. *Oncogene* **33**, 5274-5287 (2014).
26 Motaghed, M., Al-Hassan, F. M. & Hamid, S. S. Thymoquinone regulates gene expression levels in the estrogen metabolic and interferon pathways in MCF7 breast cancer cells. *Int J Mol Med* **33**, 8-16 (2014).
27 Reich, N. C. A death-promoting role for ISG54/IFIT2. *J Interferon Cytokine Res* **33**, 199-205 (2013).
28 Huber-Keener, K. J. *et al.* Differential gene expression in tamoxifen-resistant breast cancer cells revealed by a new analytical model of RNA-Seq data. *PLoS One* **7**, e41333 (2012).
29 Scurr, L. L. *et al.* Ankyrin repeat domain 1, ANKRD1, a novel determinant of cisplatin sensitivity expressed in ovarian cancer. *Clin Cancer Res* **14**, 6924-6932 (2008).
30 Dago, D. N. *et al.* Estrogen receptor beta impacts hormone-induced alternative mRNA splicing in breast cancer cells. *BMC Genomics* **16**, 367 (2015).
31 Figueiredo, N. B. *et al.* Estrogen-responsive genes overlap with triiodothyronine-responsive genes in a breast carcinoma cell line. *ScientificWorldJournal* **2014**, 969404 (2014).
32 Saletta, F., Suryo Rahmanto, Y., Noulsri, E. & Richardson, D. R. Iron chelator-mediated alterations in gene expression: identification of novel iron-regulated molecules that are molecular targets of hypoxia-inducible factor-1 alpha and p53. *Mol Pharmacol* **77**, 443-458 (2010).
33 Zhang, X. *et al.* A pituitary-derived MEG3 isoform functions as a growth suppressor in tumor cells. *J Clin Endocrinol Metab* **88**, 5119-5126, (2003).

Supplementary Table S4. Genes up and down regulated of top five canonical pathways (IPA)

Pathways	Up-regulated	Down-regulated	Total
1.EIF2 signaling	**AKT1, PPP1CA,** **EIF** (1/2AK1/2B2/2B5/3B/3C/3D/3G/3I/3K/3L/4A1-4A3/4G1) **RPL** (3-8/11-14/17-19/22-24/ 26-27/29-32/34-39/41/10A/ 13A/18A/23A/26L1/35A/36AL/ 37A/7A/P0-P2) **RPS** (2/3/5-9/11-16/18-21/23/25/27-30/15A/3A/4X/A)	**PIK3CA** and **RRAS2**	87
2.Mitochondrial dysfunction	*Include all genes the oxidative phosphorylation more:* **AIFM1**, **APH1A**, **FIS1**, **GPX4**, **HSD17B10**, **PARK7**, **PRDX** (3 and 5) and **TXN2**	**BACE2**, **BCL2**, **NDUFAF2** and **MAP2K4**	55
3.mTOR signaling	**AKT1**, **DDIT4**, **RND3**, **EIF** (3B/3C/3D/3G/3I/3K/3L/4A1-4A3/4EBP1/4G1) **PPP2** (R4 and R1A), **RAC1**, **RHO** (A and C), **VEGFA** and *include all the genes* **RPS** *of pathway* EIF2 *signaling*	**PIK3CA**, **RICTOR**, **RPS6KA3** and **RRAS2**	52
4.Regulation of EIF4 and p70S6K	**AKT1**, **PPP2** (R4 and R1A), **EIF4EBP1** more *include all the genes* **EIF** (*except* EIF2AK1) *and* **RPS** *of pathway* EIF2 *signaling*	**ITGA2**, **PIK3CA** and **RRAS2**	48
5.Oxidative phosphorylation	**ATP** (5B/5C1/5E/5G1/5H/5I/ 5J2/5L/5O) **NDUF** (A1/A2/A6/A9/A13/B1-B3/B7-B11/S2-S3/S5/S8/V1) **COX** (4I1/5A/5B/6A1/6B1/7A2/ 7B/7C/8A), **CYB5A**, **CYC1**, **SDHB** and **UQCR** (C1 and H)	**NDUFS4**	42

Supplementary Table S5. Canonical pathways related to cellular cycle (IPA)

**Pathways**	**Up-regulated**	**Down-regulated**	**Total**
PI3K/AKT signaling	AKT1, CDC37, CDKN1B, EIF4EBP1, HSP90AB1, HSP90B1, INPPL1, PPP2R4, PPP2R1A and TP53	BCL2, ITGA2, PIK3CA and RRAS2	14
PTEN signaling	AKT1, CDKN1B, INPPL1, MCRS1 and PIK3CA	BCL2, BMPR2, ITGA2, MAGI3, RAC1 and RRAS2	11
Cell cycle: G1/S Checkpoint regulation	CDK4, CDKN1B, E2F4, MAX, MYC, PA2G4, TP53, RPL5 and RPL11		9
HER-2 signaling in breast Cancer	AKT1, CDKN1B, ITGB5 and TP53	AREG, ITGB6, PARD3, PIK3CA and RRAS2	9
Cell cycle regulation by BTG family protein	CDK4, E2F4, PPP2R4, PPP2R1A and PRMT1		5
Estrogen-mediated S-phase entry	CDK4, CDKN1B, E2F4 and MYC		4

Supplementary Table S6. Relations between lncRNA and the a adjacent mRNA protein-coding gene differentially expressed.

Symbol mRNA	Gene name	Fold change	Symbol lncRNA	Fold change	Location lncRNA	Location mRNA	Chr
ABHD2	Abhydrolase domain containing 2	2.5	ABHD2-1:5	**	89584454-89626050	89631381-89745591	15
ADI1	Acireductone dioxygenase 1	3.0	ADI1-2:1	**	3302112-3305236	3501690-3523350	2
ANKRD30A	Ankyrin repeat domain 30A	*	ANKRD30A-4:1	*	37229037-37232860	37414785-37521495	10
ANKRD40	Ankyrin repeat domain 40	2.2	ANKRD40-1:1<br>ANKRD40-1:5	*<br>*	48840212-48844918<br>48838535-48844877	48770551-48785270	17<br>17
ARL15	ADP-ribosylation factor-like 15	-4.6	ARL15-1:1	*	53072474-53115516	53180614-53606403	5
ARL4A	ADP-ribosylation factor-like 4A	-2.0	ARL4A-1:1	*	13141016-13142014	12726452-12730559	7
ARMCX3	Armadillo repeat containing, X-linked 3	2.1	ARMCX3-1:1	2.7	100882858-100883508	100878120-100882831	X
ATF7IP	Activating transcription factor 7 interacting protein	-3.2	ATF7IP-3:1	**	14369524-14374231	14518611-14651697	12
BOD1	Biorientation of chromosomes in cell division 1	2.7	BOD1-2:1	*	173217812-173236042	173034148-173043666	5
CCT5	Chaperonin containing TCP1, subunit 5 (epsilon)	2.2	CCT5-2:1	-2.5	10137250-10138477	10250282-10266501	5
CD63	CD63 molecule	2.1	CD63-1:1	6.0	56155334-56156412	56119230-56122910	12
CHD2	Chromodomain helicase DNA binding protein 2	-2.4	CHD2-4:2	-2.5	93855786-94098099	93443551-93571237	15
CHMP1A	Chromatin modifying protein 1A	2.6	CHMP1A-2:1	*	89458783-89478972	89710843-89724129	16
COMMD10	COMM domain containing 10	-2.4	COMMD10-3:1	**	115910178-115927094	115420727-115628978	5
COMMD3	COMM domain containing 3	2.1	COMMD3-1:1	*	22546715-22547477	22605312-22609246	10
CYB5A[4]	Cytochrome b5 type A (microsomal)	2.6	CYB5A-1:2	*	71878626-71879804	71920527-71959251	18
DAD1	Defender against cell death 1	2.4	DAD1-2:1<br>DAD1-2:6	**<br>**	22849083-22951948<br>22850773-22901719	23033807-23058143	14
EEF1B2	Eukaryotic translation elongation factor 1 beta 2	5.4	EEF1B2-2:2	**	206950246-206950880	207024318-207027653	2
EIF3B[1]	Eukaryotic translation initiation factor 3, subunit B	3.1	EIF3B-1:1	**	2423756-2428232	2394474-2420380	7
EIF4A3[1]	Eukaryotic translation initiation factor 4A, isoform 3	2.3	EIF4A3-1:1	4.0	77965743-77966640	78109013-78120982	17
FAM83B	Family with sequence similarity 83, member B	-2.4	FAM83B-1:1	2.3	54807965-54809897	54711569-54806820	6
GAPDH	Glyceraldehyde-3-phosphate dehydrogenase-like 6	2.7	GAPDH-2:1	**	6619441-6619688	6643657-6647536	12
GGCT	Gamma-glutamyl cyclotransferase	3.2	GGCT-1:21	-7.0	30555925-30602441	30536237-30544457	7
GLCE	Glucuronic acid epimerase	-2.0	GLCE-1:1	**	69571014-69580903	69452973-69564544	15
H3F3A	H3 histone family 3A	-2.9	H3F3A-1:1	*	226274582-226277993	226250408-226259703	1
HIATL1	Hippocampus abundant transcript-like 1	-2.5	HIATL1-4:1	3.9	97320996-97330312	97136833-97223202	9
HIST1H2AG	Histone cluster 1, H2AG	4.5	HIST1H2AG-2:1	8.0	26988232-26991703	27100817-27101314	6
ITGA2[7]	Integrin, alpha 2 (CD49B, alpha 2 subunit of VLA-2 receptor)	-2.3	ITGA2-1:3	*	52405707-52410027	52285156-52390609	5
LEMD3	LEM domain containing 3	-2.2	LEMD3-1:1	**	65675437-65680508	65563351-65642141	12
LRBA	LPS-responsive vesicle trafficking, beach and anchor containing	-2.3	LRBA-2:1	-3.5	150509142-150817385	151185594-151936649	4
MYC[3]	v-myc myelocytomatosis viral oncogene homolog (avian)	4.4	MYC-2:24<br>MYC-2:5<br>MYC-2:8<br>MYC-2:9	*<br>**<br>4.5<br>2.2	129001463-129108824<br>128806806-128951855<br>128808059-128903041<br>128808066-128903206	128748315-128753680	8
NRIP1	Nuclear receptor interacting protein 1	-2.8	NRIP1-2:1	2.7	16742841-16774624	16333556-16437126	21
NT5DC1	5'-nucleotidase domain containing 1	-2.7	NT5DC1-1:1	4.0	116575370-116577906	116421999-116566853	6
PARK7[6]	Parkinson disease (autosomal recessive, early onset) 7	2.8	PARK7-1:1	*	8086798-8182762	8021714-8045342	1
PCBP1	Poly(rC) binding protein 1	3.0	PCBP1-1:1	**	70316853-70323232	70314585-70316335	2
PGR	Progesterone receptor	-14.0	PGR-1:1	**	100554893-100558686	100900355-101000544	11
PGRMC1	Progesterone receptor membrane component 1	2.6	PGRMC1-1:1	2.1	118425492-118469573	118370211-118378429	X
PLCB1	Phospholipase C, beta 1 (phosphoinositide-specific)	-4.6	PLCB1-1:1	**	8000549-8008603	8113296-8865547	20
PMP22	Peripheral myelin protein 22	18.4	PMP22-1:1	**	15170778-15175607	15133096-15168644	17

PPP2R4[5]	Protein phosphatase 2A activator, regulatory subunit 4	3.7	PPP2R4-4:1<br>PPP2R4-5:1<br>PPP2R4-5:3<br>PPP2R4-5:4	-3.1<br>*<br>-5.4<br>-3.0	132083295-132087184<br>132094579-132101180<br>132099158-132109743<br>132104122-132121817	131873228-131911225	9
PSME1	Proteasome (prosome, macropain) activator subunit 1 (PA28 alpha)	3.0	PSME1-1:2	3.1	24608654-24609653	24605378-24608176	14
RASA1	RAS p21 protein activator (GTPase activating protein) 1	-3.0	RASA1-3:4<br>RASA1-3:9	4.0<br>3.8	87564841-87581884<br>87564864-87583473	86564151-86687733	5
RPL24[2]	Ribosomal protein L24	4.0	RPL24-2:3	**	102382007-102392757	101399934-101405563	3
RPLP1[2]	Rbosomal protein, large, P1	4.4	RPLP1-1:1<br>RPLP1-1:11<br>RPLP1-1:18<br>RPLP1-1:9	**<br>**<br>10.0<br>**	69755365-69857576<br>69854062-69863422<br>69857531-69863775<br>69850531-69863422	69745159-69747884	15
RPS14[1]	Ribosomal protein S14	4.5	RPS14-1:2	**	149855094-149865531	149823792-149829319	5
RPS7[1]	Ribosomal protein S7	2.3	RPS7-1:10	2.3	3606168-3609321	3622853-3628509	2
SBDS	Shwachman-Bodian-Diamond syndrome pseudogene	2.5	SBDS-5:1<br>SBDS-5:2	-4.0<br>-2.9	65956996-65958553<br>65956083-65958525	66452690-66460588	7
SCD	Stearoyl-CoA desaturase (delta-9-desaturase)	7.0	SCD-1:10	2.0	102133372-102143125	102106772-102124588	10
SLC12A2	Solute carrier family 12 (sodium/potassium/chloride transporters), member 2	3.3	SLC12A2-1:2	**	127270935-127277199	127419483-127525380	5
SLC16A4	Solute carrier family 16, member 4 (monocarboxylic acid transporter 5)	-2.5	SLC16A4-1:1	6.0	110828997-110881793	110905473-110933704	1
SLC25A5	Solute carrier family 25 (mitochondrial carrier; adenine nucleotide translocator), member 5	3.2	SLC25A5-1:1	**	118556880-118557510	118602363-118605359	X
SLITRK6	SLIT and NTRK-like family, member 6	-2.1	SLITRK6-9:4	*	88096247-88323218	86366922-86373483	13
SSR3	Signal sequence receptor, gamma (translocon-associated protein gamma)	2.9	SSR3-3:1<br>SSR3-3:2	**<br>2.2	156470852-156534851<br>156465135-156534823	156257929-156272973	3
SUMF2	Sulfatase modifying factor 2	3.3	SUMF2-2:1	**	56560817-56564978	56131917-56148365	7
TCF12	Transcription factor 12	-2.7	TCF12-1:13	*	57592563-57599959	57210833-57580716	15
TMEM167B	Transmembrane protein 167B	2.5	TMEM167B-1:3	2.3	109642815-109643234	109633403-109639554	1
TOB1	Transducer of ERBB2, 1	2.1	TOB1-2:1	*	48986998-48987593	48939584-48941413	17
TRMT112	tRNA methyltransferase 11-2 homolog (S. cerevisiae); similar to CG12975	4.4	TRMT112-2:1	*	64161814-64163303	64084163-64085033	11
USP3	Ubiquitin specific peptidase 3	-2.5	USP3-1:2	2.4	63682429-63729347	63796810-63883663	15
WDR1	WD repeat domain 1	2.3	WDR1-1:1	5.0	10069713-10074643	10075963-10118573	4
WDR74	WD repeat domain 74	2.6	WDR74-1:1<br>WDR74-1:12<br>WDR74-1:14<br>WDR74-1:22<br>WDR74-1:25<br>WDR74-1:4	9.3<br>6.0<br>2.5<br>2.5<br>-2.3<br>-2.3	62619730-62623386<br>62621840-62622960<br>62620826-62622960<br>62619460-62622708<br>62619520-62621168<br>62619460-62623339	62600383-62607628	11
ZNF259	Zinc finger protein 259	2.2	ZNF259-1:1	-2.5	116645826-116646592	116649276-116658739	11
ZNF740	Zinc finger protein 740	2.0	ZNF740-1:1	**	53553370-53554784	53574535-53584654	12

* Only expressed in monolayers
** only expressed in spheroids
[1,2] Genes of EIF2 signaling
[1] Genes of mTOR signaling
[1,5,7] Genes of regulation of EIF4 and p70S6K pathways
[3] Gen of cell cycle: G1/S Checkpoint and estrogen-mediated S-phase entry
[4,6] Genes of mitochondrial dysfunction and Oxidative phosphorylation pathways
[5] Genes Cell cycle by BTG,
[5,7] Genes PI3K/AKT pathways
[7] Genes of PTEN signaling

Supplementary Table S7. qRT-PCR validation of lncRNAs and mRNAs genes.

**GEN**	**Relative expression**		**p-value**	**Validation method**
	**qRT-PCR**	**RNA-seq**	**qRT-PCR**	
lnc-AGBL1-4:1	-19.69 ± 1.68	-5.1	0.001	Relative standard curve
lnc-CAMK1G-1:2	-2.77 ± 0.65	-11.3	0.001	Relative standard curve
lnc-PPP2R4-4:1	-1.57 ± 0.51	-3.1	0.042	Relative standard curve
lnc-KIAA1755-5:3	0.57 ± 0.33	-4.0	**0.265**	Relative standard curve
lnc-MLN-1:2	3.24 ± 1.12	23	0.022	Relative standard curve
lnc-IGSF5-1:1	1.75 ± 0.70	22	0.015	Relative standard curve
lnc-CPEB4-6:1	2.44 ± 1.02	19	0.030	Relative standard curve
ITGB6	0.15554072 ± 0.30	-10.52	0.00147	Comparative ΔΔ C_T
ANKRD30A	0.144032353 ± 0.23	*	0.00001	Comparative ΔΔ C_T
**MYC**	0.84319542 ± 0.22	4.4	0.0007	Comparative ΔΔ C_T
GAPDH	1.292590582 ± 0.15	2.65	**0.406**	Comparative ΔΔ C_T
p53	1.555923172 ± 0.06	2.30	0.0015	Comparative ΔΔ C_T
HIST1H2AG	1.076666667 ± 0.17	4.49	**0.06**	Comparative ΔΔ C_T
NDUFA1	3.494077936 ± 0.15	5.8	0.0008	Comparative ΔΔ C_T
COX8A	9.030698223 ± 0.16	5.74	0.013	Comparative ΔΔ C_T
lnc-GAPDH-2:1	3.07182457 ± 0.19	**	0.028	Comparative ΔΔ C_T
lnc-HIST1H2AG-2:1	3.83134634 ± 0.08	8.0	0.041	Comparative ΔΔ C_T
**lnc-MYC-2:24**<br>**2:5**<br>**2.8**<br>**2:9 isoforms**	0.23693361 ± 0.22	*<br>**<br>4.5<br>2.2	0.000004	Comparative ΔΔ C_T

Genes * only monolayer or ** only spheroids this was expressed

Supplementary Table S8. lncRNAs dysregulated with know function.

**ID lncRNA LNCipedia (Ensembl)**	**Disease**	**Description**	**Ref.**
lnc-C22orf32-1	Nasopharyngeal carcinoma (NPC)	High levels of lnc-C22orf32-1 was found in the primary NPC tissues. This increase was associated with advanced tumor stages. High levels of lnc-C22orf32-1 were associated with the male patients *The differential expression of lncRNA observed in NPC might be attributed to epigenetic regulation including microRNA, DNA methylation, and histone modification	1, 2
lnc-MYC-2 (PVT1)	Breast, Ovarian Pancreas Renal and Gastric cáncer Diabetes and other	e.g., Amplification of PVT1 contributes to the pathophysiology of ovarian and breast cancer. *The lncRNA PVT1 interacts with MYC in the nucleus.	1, 3*
lnc-CPN2-1 (LINC00887)	Renal cell carcinoma (RCC)	lnc-CPN2-1 overexpression in RCC Small interfering RNA (siRNA)-mediated knockdown of lnc-CPN2-1 did not influence cell proliferation	4
lnc-PPP2R4-5 (LINC01503) lnc-FAM46A-1 (LINC01526) lnc-MBL2-4 (LINC01468)	Tongue squamous cell carcinoma (SCC)	These lncRNAs were upregulated in the microdissected tongue SCC tissues. High level of lnc-MBL2-4:3 was associated with the node positive tongue cancer patients In addition, reduced expression of lnc-MBL2-4:3 in the cisplatin resistant HN21B cells	5
lnc-HSCB-1 (TTC28-AS1)	HSCs (hematopoietic stem cell) and limphoid progenitor cells	This lnc-RNA showed a strong co-expression with the BCR protein-conding gene. *BCR can be considered as a candidate tumor suppressor gene which may be involved in meningioma pathogenesis	6,7*
lnc-SERPINC1-1 (GAS5)	Breast,Renal cancer B-cell neoplasms *Kidney,Lymphoma Melanoma, Prostata Systemic lupus erythaematosus and autoimmune disease	e.g., GAS5 promoted the apoptosis of triple-negative and oestrogen receptor-positive cells but only dual PI3K/mTOR inhibition was able to enhance GAS5 levels in all cell types	1, 8*
Lnc-DDX27-1 (ZFAS1)	Breast and Ductal carcinoma	ZFAS1 show distinct patterns of expression during post-pubertal mammary Development. ZFAS1 is a putative tumor suppressor gene.	1, 9
lnc-C11orf89-2 (H19)	Breast, bladder, cervical, adrenocortical cancer and other	Estrogen signaling increased the expression of *H19* and ERα. H19 expression *also is* important for estrogen-induced luminal progenitor expansion. *c-Myc and H19 expression shows strong association in primary breast and lung carcinomas	1*, 10
lnc-SCYL1-1 (MALAT1)	Breast, cervical, bladder hepatocelluar	The MALAT1 level was significantly lower in tumor tissue compared to adjacent non-cancerous tissue in breast cancer. Downregulation of MALAT1 induced EMT.	11

	carcinoma and other	Induction of EMT by MALAT1 downregulation may be mediated by the PI3K-AKT pathway	
lnc-DLK1-4 (MEG3)	Breast, bladder cancer, Glioma	MEG3a exhibited a strong ability to inhibit cell growth and proliferation in several human cancer cell lines, such HeLa, MCF-7, and H4.	12
lnc-NAP1L2-1 (XIST1)	Breast, ovarian, bladder, testicular cáncer and other	XIST typically is expressed by all female somatic cells, XIST expression has been found to be lost in female breast, ovarian, and cervical cancer cell lines.	13
lnc-TMEM30B-4:1 (HIF1A-AS1)	*Non-small cell lung cancer (NSCLC), Kidney, Renal Oesophageal denocarcinoma and Cardiovascular disease	*The expression of XIST and HIF1A-AS1 in tumor tissues and serum of NSCLC was higher than that of the control group (nontumourous)	1, 14
lnc-ATXN7-8 (ADAMTS9-AS2)	Glioma	ADAMTS9-AS2 was downregulated in glioma tissue. Co-relationship of ADAMTS9-AS2 with ADAMTS9 and DNMT1.<br>Overexpression of ADAMTS9-AS2 inhibited migration and invasion abilities of the T98G cells While knockdown opposite effect in U87 cells	15
lnc-TMEM60-1 (APTR)	Glioblastoma	*APTR* represses *p21 t*ranscription by recruiting the PRC2 complex to the *p21* promoter.<br>*APTR* is important for G1-S transition most likely due to a requirement for the activity of CyclinE/CDK2 kinase	16
lnc-METTL15-4<br><br>(BDNF-AS)	Huntington's disease (HD) Obesity, Schizophrenia Other	*BDNF*-AS and *BDNF* transcripts are Co-expressed in many tissues, which suggest *BDNF*-AS potential for regulation of *BDNF* mRNA in monkey and mouse tissues<br>*BDNF-AS inhibits BDNF transcription by recruiting EZH2 to the BDNF promoter region and plays an important role in the development of HD	1, 17
lnc-EMX2-4 (CASC2)	Glioma, endometrial, colorectal cáncer snd Melanoma, renal carcinoma	CASC2 was lowly expressed in glioma tissues. Overexpression of CAS2 inhibited proliferation, migration, and invasion, and promoted cell apoptosis.<br>Up-regulated CASC2 decreased the expression of miR-2.	1,18
lnc-RPLP1-1 (DRAIC)	Prostate cancer	Prostate cancer is accompanied by a decrease of FOXA1 and NKX3-1, which leads to the decrease of tumor-suppressive lncRNAs, DRAIC and PCAT29.	19
lnc-CHIC1-3 (JPX)	Embryonic stem cell (ES)	*Jpx* expression increases during ES cell differentiation and remained elevated in somatic cells<br>*Xist* is controlled by two parallel RNA switches – Tsix for Xa and Jpx for Xi. Whereas Tsix represses *Xist* on Xa, Jpx activates *Xist* on Xi	20
lnc-LPAR6-1 (LINC00441)	Lung carcinoma cell	A549 cells shows a predominantly nuclear localization of ncRNA-RB1 (LINC00441). This lncRNA no effects on RB1 transcript or protein levels, suggesting that the two transcripts do not regulate each other.Transcriptional regulation of CALR expression by ncRNA-RB1	21
lnc-MAF-2 (MAFTRR)	T and B lymphocyte	High expression of linc-MAF-4 correlated with a low amount of *MAF* transcript in $CD4^+$ T_H1 cells, conversely T_H2 cells had low expression of linc-MAF-4 and abundant *MAF* transcripts.	22

lnc-TRAF5-1<br><br>(LINC00467)	Neuroblastoma	Linc00467 suppressed the expression of its downstream protein-coding gene RD3, and induced neuroblastoma cell survival by reducing the expression of the tumour suppressor gene DKK1<br>Down-regulation of N-Myc expression resulted in increased linc00467 expression.	23
lnc-YBEY-2 (MCM3AP-AS1)	Human B-cell	This lncRNA were enriched in the nucleus and were upregulated in high MYC expression.<br>MYC binding was enriched at the promoter of these lncRNA	24
lnc-NEDD1-4 (RMST)	Brain<br>*Rhabdomyosarcoma	*RMST* associates with SOX2, one of the most important transcription factors controlling neural stem cell (NSC) fate<br>Knockdown of *RMST* prevented neuronal differentiation	1*, 25
lnc-ABCB4-1 (TP53TG1)	Colon cancer	Under conditions of cellular stress (ultraviolet irradiation or exposure to bleomycin or cisplatin), expression of TP53TG1 was induced in a wild-type TP53-dependent manner	26
lnc-OSBP2-2 (TUG1)	MCF-7 and HeLa cells<br>B-cell neoplasms<br>bladder cáncer<br>non-small cell lung cancer<br>Huntington's disease	*Under genotoxic stress-induced apoptosis in HeLa and MCF-7 cells TUG1, UCA1, and PANDA not affected.<br>Cell proliferation inhibition and apoptosis induction were observed in TUG1 siRNA-transfected bladder urothelial carcinoma T24 and 5637 cells.	1, 27
lnc-HEATR6-1 (WFDC21P)	Dendritic cells	lnc-D (WFDC21P), was exclusively expressed in human conventional dendritic cells (DCs).<br>Knockdown of lnc-DC impaired DC differentiation from human monocytes and mouese bone marrow cells and reduced capacity of DCs to stimulate T cell activation. Lnc-DC mediated these effects by activating the transcription factor STAT3.	28
lnc-TGFBR2-1 (RP11-1024P17.1)	Vascular disease.	This lncRNA could to be implicated in proliferation and migration of vascular smooth muscle cells (VSMCs)	29

References.

1 Chen, G. *et al*. LncRNADisease: a database for long-non-coding RNA-associated diseases. *Nucleic Acids Res* **41**, D983-986, doi:10.1093/nar/gks1099 (2013).

2 Gao, W., Chan, J. Y. & Wong, T. S. Differential expression of long noncoding RNA in primary and recurrent nasopharyngeal carcinoma. *Biomed Res Int* **2014**, 404567 (2014).

3 Colombo, T., Farina, L., Macino, G. & Paci, P. PVT1: a rising star among oncogenic long noncoding RNAs. *Biomed Res Int* **2015**, 304208 (2015).

4 Deng, M. *et al*. Identification of novel differentially expressed lncRNA and mRNA transcripts in clear cell renal cell carcinoma by expression profiling. *Genom Data* **5**, 173-175 (2015).

5 Gao, W., Chan, J. Y. & Wong, T. S. Long non-coding RNA deregulation in tongue squamous cell carcinoma. *Biomed Res Int* **2014**, 405860 (2014).

6 Wozniak, K. *et al*. BCR expression is decreased in meningiomas showing loss of heterozygosity of 22q within a new minimal deletion region. *Cancer Genet Cytogenet* **183**, 14-20 (2008).

7 Casero, D. *et al*. Long non-coding RNA profiling of human lymphoid progenitor cells reveals transcriptional divergence of B cell and T cell lineages. *Nat Immunol* **16**, 1282-1291 (2015).

8 Qiao, H. P., Gao, W. S., Huo, J. X. & Yang, Z. S. Long non-coding RNA GAS5 functions as a tumor suppressor in renal cell carcinoma. *Asian Pac J Cancer Prev* **14**, 1077-1082 (2013).

9 Shore, A. N., Herschkowitz, J. I. & Rosen, J. M. Noncoding RNAs involved in mammary gland development and tumorigenesis: there's a long way to go. *J Mammary Gland Biol Neoplasia* **17**, 43-58 (2012).

10 Basak, P. *et al*. Estrogen regulates luminal progenitor cell differentiation through H19 gene expression. *Endocr Relat Cancer* **22**, 505-517 (2015).

11 Xu, S. *et al*. Downregulation of long noncoding RNA MALAT1 induces epithelial-to-mesenchymal transition via the PI3K-AKT pathway in breast cancer. *Int J Clin Exp Pathol* **8**, 4881-4891 (2015).

12 Zhang, X. *et al*. A pituitary-derived MEG3 isoform functions as a growth suppressor in tumor cells. *J Clin Endocrinol Metab* **88**, 5119-5126 (2003).

13 Weakley, S. M., Wang, H., Yao, Q. & Chen, C. Expression and function of a large non-coding RNA gene XIST in human cancer. *World J Surg* **35**, 1751-1756 (2011).

14 Tantai, J., Hu, D., Yang, Y. & Geng, J. Combined identification of long non-coding RNA XIST and HIF1A-AS1 in serum as an effective screening for non-small cell lung cancer. *Int J Clin Exp Pathol* **8**, 7887-7895 (2015).

15 Yao, J. *et al*. A new tumor suppressor LncRNA ADAMTS9-AS2 is regulated by DNMT1 and inhibits migration of glioma cells. *Tumour Biol* **35**, 7935-7944 (2014).

16 Negishi, M. *et al*. A new lncRNA, APTR, associates with and represses the CDKN1A/p21 promoter by recruiting polycomb proteins. *PLoS One* **9**, e95216 (2014).

17 Modarresi, F. *et al*. Inhibition of natural antisense transcripts in vivo results in gene-specific transcriptional upregulation. *Nat Biotechnol* **30**, 453-459 (2012).

18 Wang, P. *et al*. Long non-coding RNA CASC2 suppresses malignancy in human gliomas by miR-21. *Cell Signal* **27**, 275-282 (2015).

19 Sakurai, K., Reon, B. J., Anaya, J. & Dutta, A. The lncRNA DRAIC/PCAT29 Locus Constitutes a Tumor-Suppressive Nexus. *Mol Cancer Res* **13**, 828-838 (2015).

20 Tian, D., Sun, S. & Lee, J. T. The long noncoding RNA, Jpx, is a molecular switch for X chromosome inactivation. *Cell* **143**, 390-403 (2010).

21 Musahl, A. S. *et al*. A long non-coding RNA links calreticulin-mediated immunogenic cell removal to RB1 transcription. *Oncogene* **34**, 5046-5054 (2015).

22 Ranzani, V. *et al*. The long intergenic noncoding RNA landscape of human lymphocytes highlights the regulation of T cell differentiation by linc-MAF-4. *Nat Immunol* **16**, 318-325 (2015).

23 Atmadibrata, B. *et al*. The novel long noncoding RNA linc00467 promotes cell survival but is down-regulated by N-Myc. *PLoS One* **9**, e88112 (2014).

24 Hart, J. R., Roberts, T. C., Weinberg, M. S., Morris, K. V. & Vogt, P. K. MYC regulates the non-coding transcriptome. *Oncotarget* **5**, 12543-12554 (2014).

25 Ng, S. Y., Bogu, G. K., Soh, B. S. & Stanton, L. W. The long noncoding RNA RMST interacts with SOX2 to regulate neurogenesis. *Mol Cell* **51**, 349-359 (2013).

26 Takei, Y., Ishikawa, S., Tokino, T., Muto, T. & Nakamura, Y. Isolation of a novel TP53 target gene from a colon cancer cell line carrying a highly regulated wild-type TP53 expression system. *Genes Chromosomes Cancer* **23**, 1-9 (1998).
27 Ozgur, E. *et al*. Differential expression of long non-coding RNAs during genotoxic stress-induced apoptosis in HeLa and MCF-7 cells. *Clin Exp Med* **13**, 119-126 (2013).
28 Wang, P. *et al*. The STAT3-binding long noncoding RNA lnc-DC controls human dendritic cell differentiation. *Science* **344**, 310-313 (2014).
29 Li, J. *et al*. A bioinformatics method for predicting long noncoding RNAs associated with vascular disease. *Sci China Life Sci* **57**, 852-857 (2014).

Supplementary Table S9. qPCR quantification for transfection of lncRNAs and the relationship of lncRNA overexpressed with its nearby coding gene (mRNA).

**lncRNAs neighbor mRNA**	lncRNAs **Fold difference (log_2)**		p-value	mRNAs **Fold difference (log_2)**		p-value
lncRNA / mRNA	*VEC -*	*VEC+*		*VEC -*	*VEC +*	
lnc-GAPDH-2:1/ mRNA GADPH	-5.75 ± 0.23	5.82 ± 0.16	0.00006	-1.77 ± 0.19	1.78 ± 0.17	0.000007
lnc-HIST1H2AG-2:1/ mRNA HIST1H2AG	-14.68 ± 0.30	15.00 ± 0.22	0.00007	-1.90 ± 0.22	2.05 ± 0.16	0.00026
lnc-MYC-2:24, 2:5, 2.8,2:9 isoforms/ mRNA MYC	-13.44 ± 0.21	13.38 ± 0.36	0.00002	-1.08 ± 0.26	2.70 ± 0.24	0.00045

VEC- Empty vector and VEC + plasmid transfection with lncRNAs

A **B**

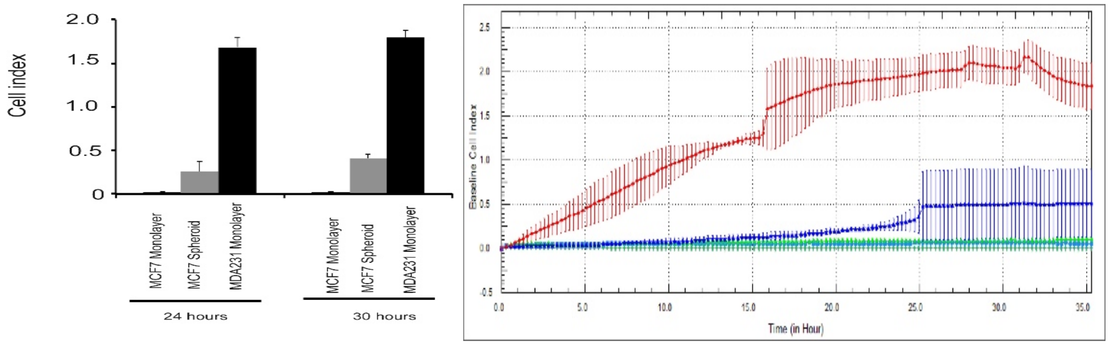

Supplementary Figure S1. Real time MCF-7 monolayers and spheroids disaggregated migration data. **(A)** Results from an RTCA migration assay conducted with FBS in the bottom chamber of a CIM plate well. MCF-7 monolayers cell and spheroids disaggregated cells migration is compared to MDA-MB-231 (Positive control). Results are shown as mean ± SD Cell Index from triplicate wells at the 24 and 30 h time point **(B)** xCELLigence plots representative. MDA-MB-231 in red, spheroids disaggregated in blue and monolayers cell in green, Light colored lines representing conducted without SBF.

A

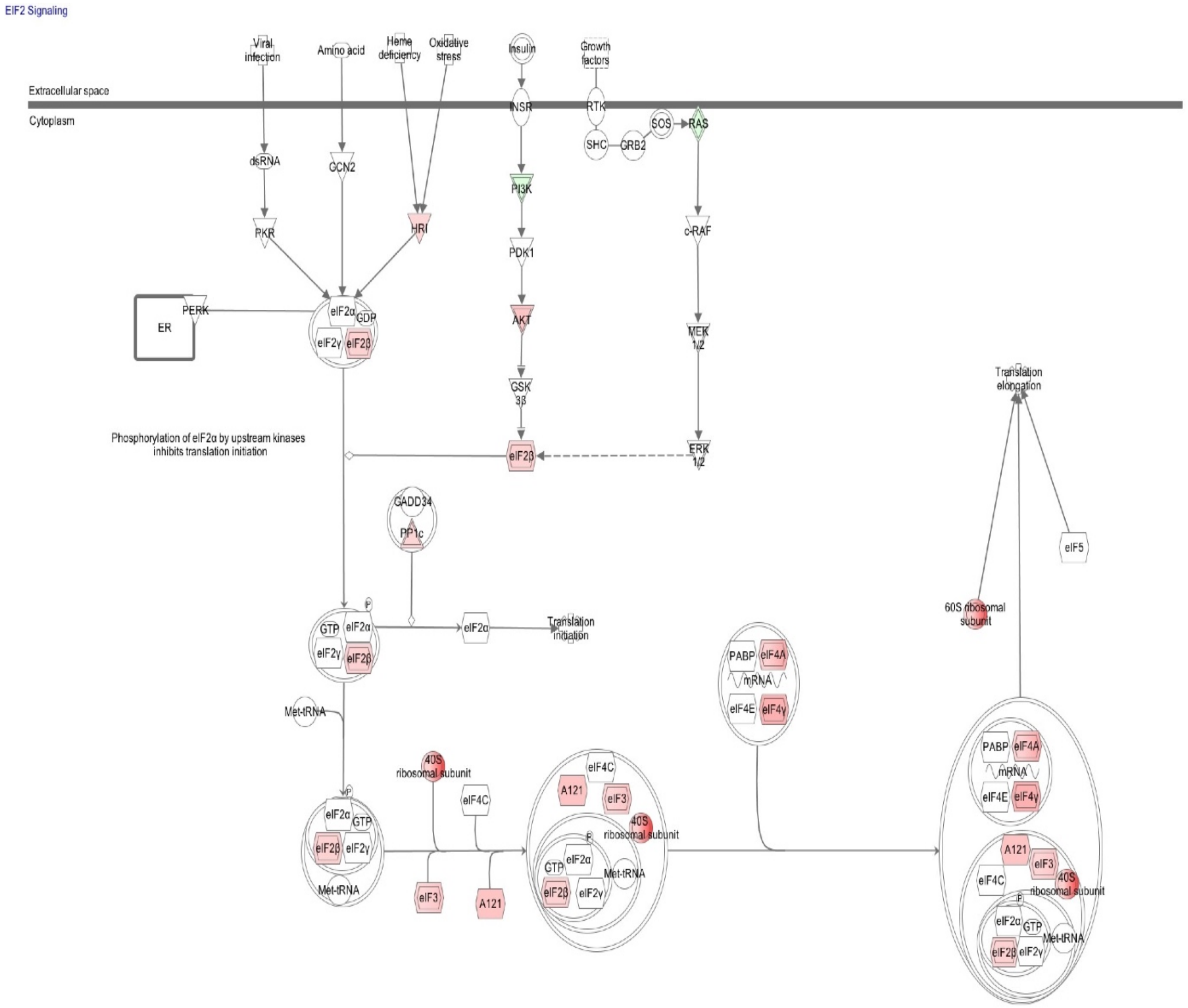

© 2000-2015 QIAGEN. All rights reserved.

B

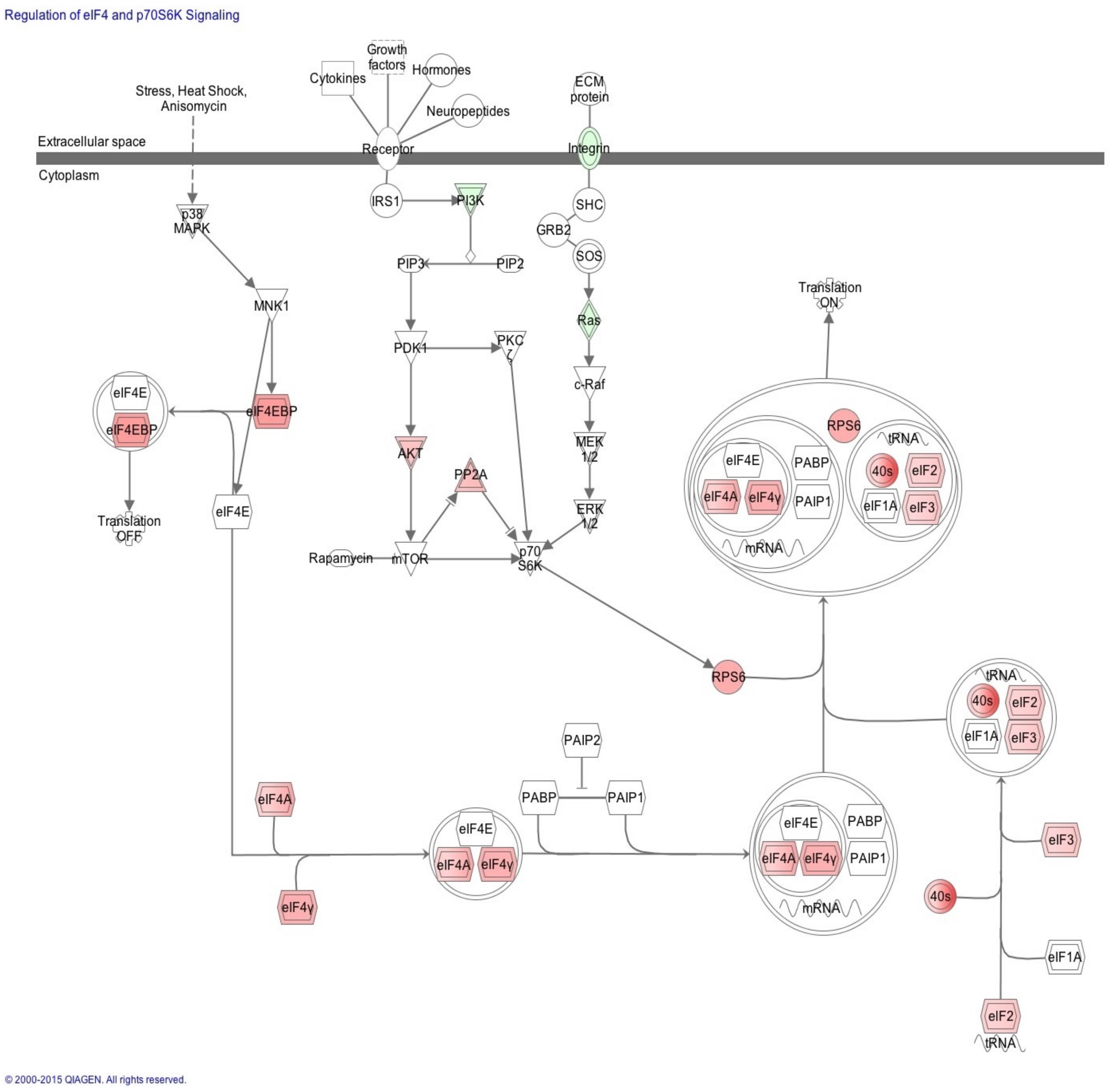
Regulation of eIF4 and p70S6K Signaling
Stress, Heat Shock, Anisomycin
Cytokines
Growth factors
Hormones
Neuropeptides
ECM protein
Extracellular space
Receptor
Integrin
Cytoplasm
IRS1
PI3K
SHC
GRB2
SOS
p38 MAPK
PIP3
PIP2
Ras
MNK1
PDK1
PKC ζ
c-Raf
Translation ON
eIF4E
eIF4EBP
AKT
MEK 1/2
RPS6
tRNA
PABP
40s
eIF2
PP2A
eIF4A
eIF4γ
PAIP1
eIF1A
eIF3
ERK 1/2
Translation OFF
mRNA
Rapamycin
mTOR
p70 S6K
PAIP2
© 2000-2015 QIAGEN. All rights reserved.

C

mTOR Signaling

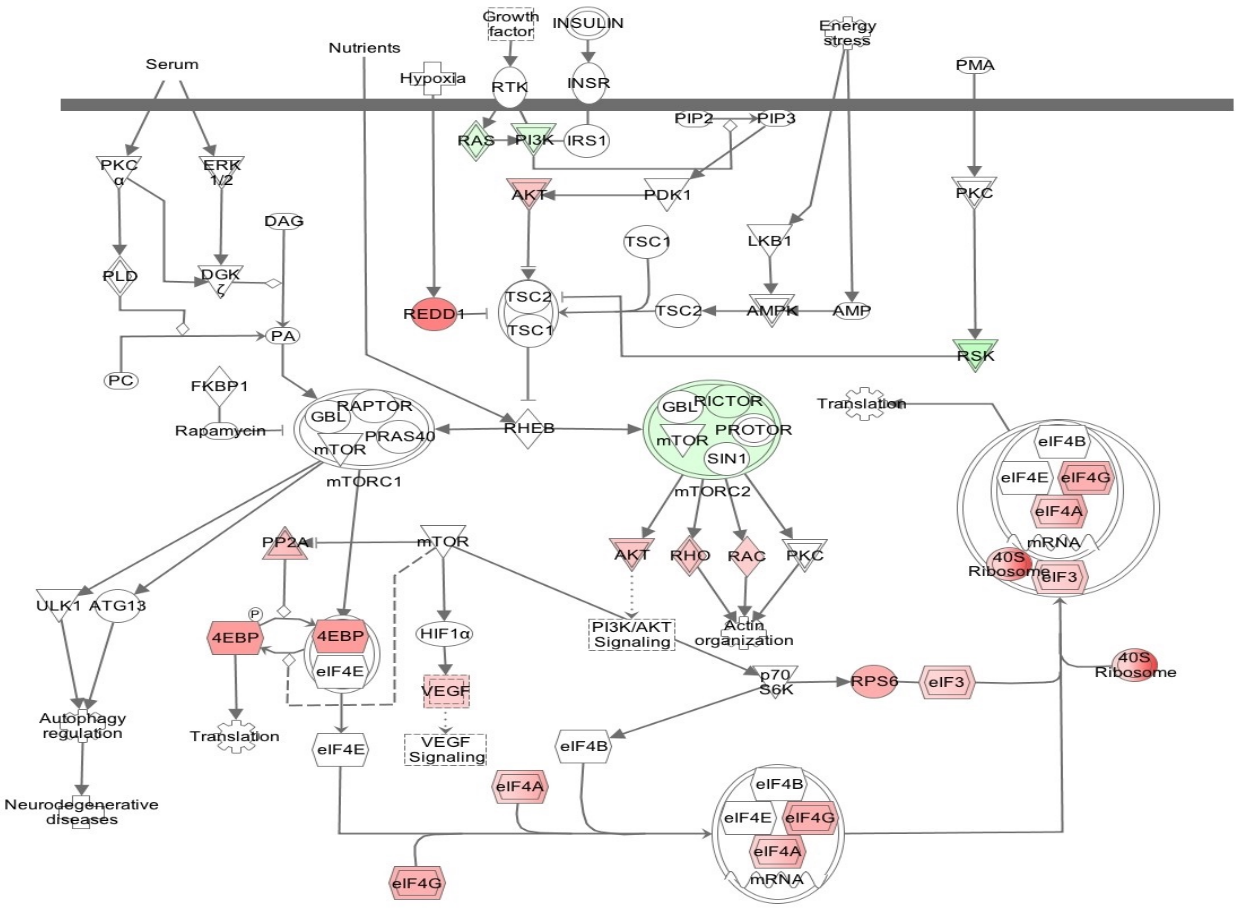

© 2000-2015 QIAGEN. All rights reserved.

D

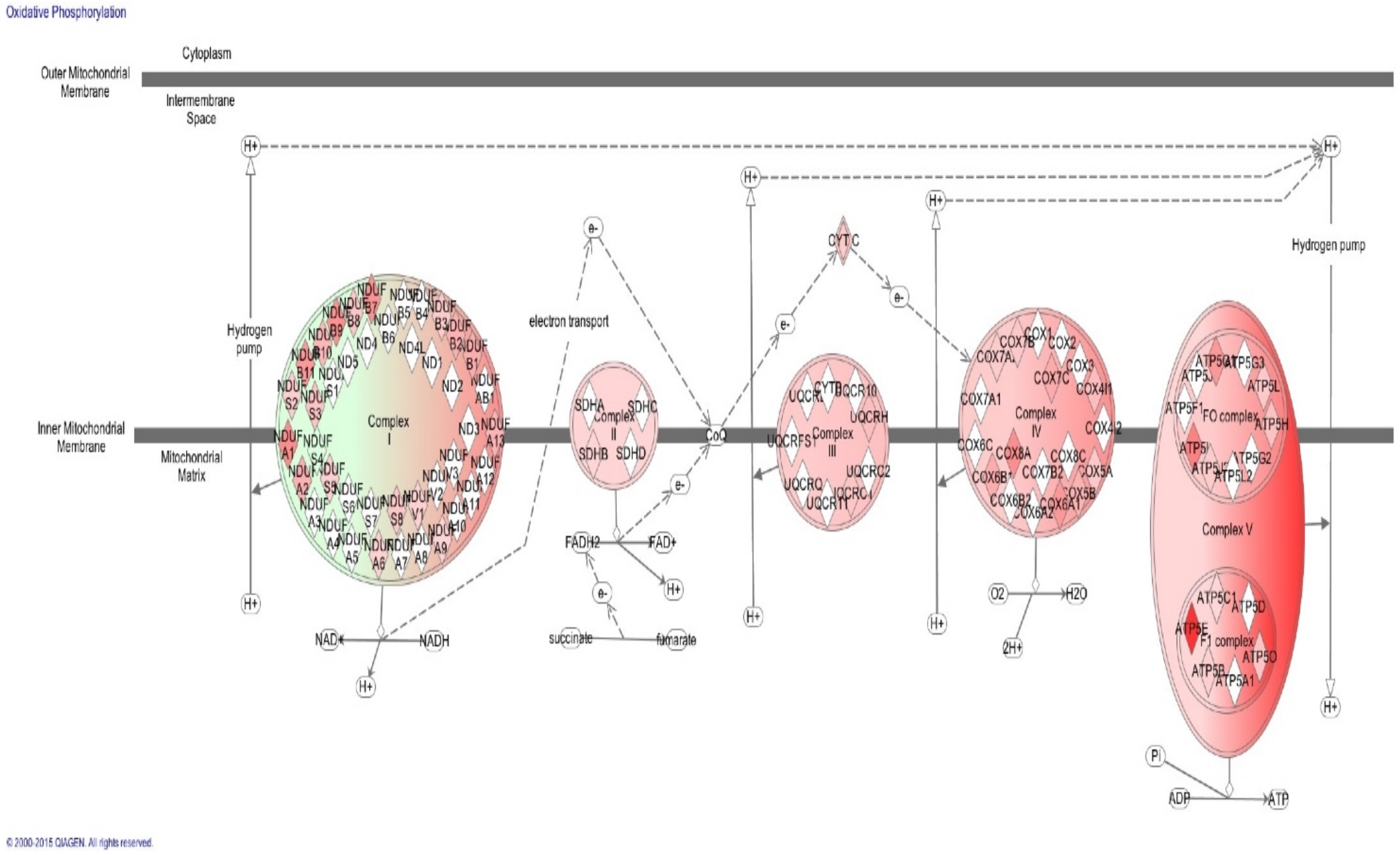
Oxidative Phosphorylation
Cytoplasm
Outer Mitochondrial Membrane
Intermembrane Space
Inner Mitochondrial Membrane
Mitochondrial Matrix
Hydrogen pump
electron transport
Complex I
Complex II
Complex III
Complex IV
Complex V
FO complex
F1 complex
CoQ
CYT C
NAD+
NADH
FADH2
FAD+
succinate
fumarate
O2
H2O
2H+
Pi
ADP
ATP
SDHA
SDHB
SDHC
SDHD
UQCRFS1
UQCRQ
UQCRH
UQCRC2
COX7A1
COX6C
COX8A
COX7B2
COX8C
COX5A
COX5B
COX6A1
COX6B2
COX4I1
COX4I2
COX3
COX2
COX1
COX7C
ATP5G3
ATP5L
ATP5H
ATP5G2
ATP5L2
ATP5F1
ATP5C1
ATP5D
ATP5E
ATP5O
ATP5B
ATP5A1
© 2000-2015 QIAGEN. All rights reserved.

E

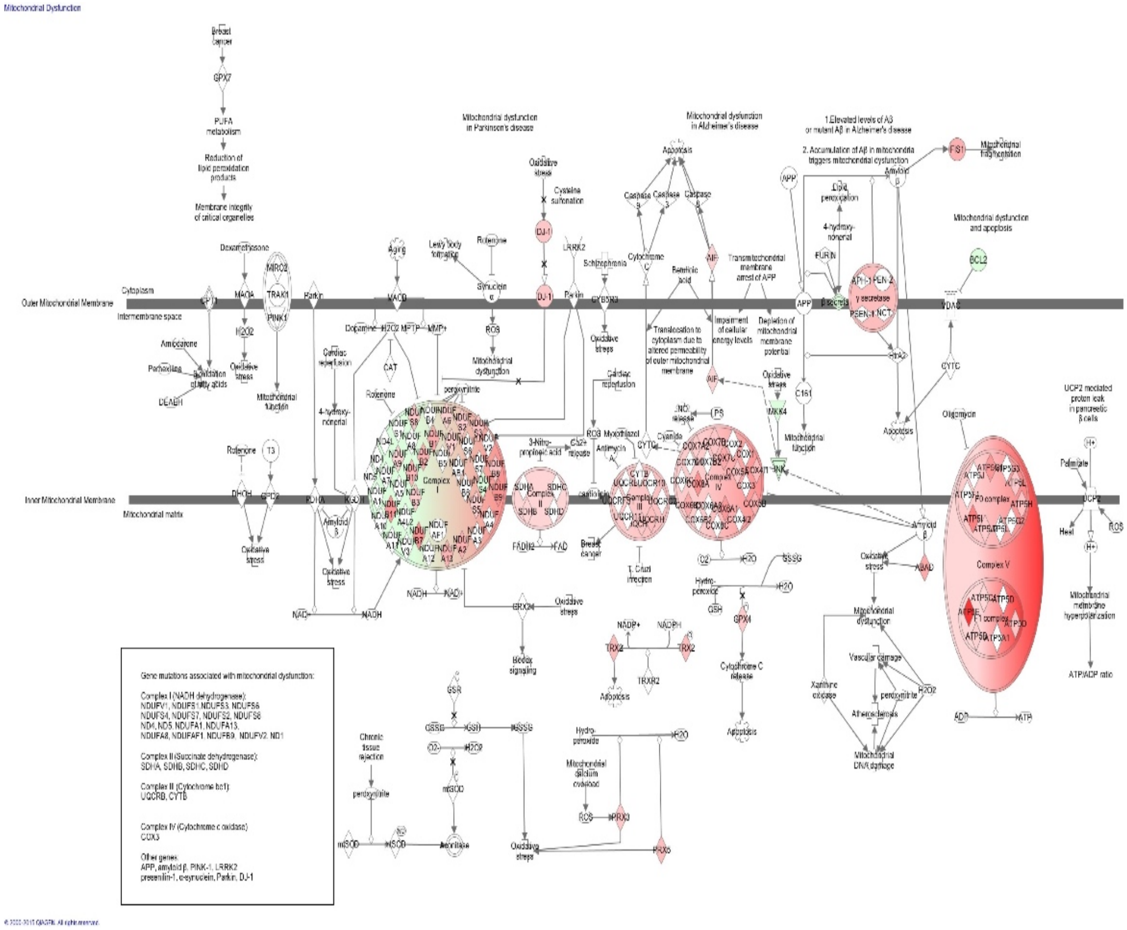

Supplementary Figure S2. The top five dysregulated canonical pathways (IPA). (A) EIF2 Signaling, (B) Regulation of elF4 and p70S6K Signaling, (C) mTOR Signaling, (D) Oxidative Phosphorylation and (E) Mitochondrial Dysfunction. *Relationships, molecules shapes and genes with synonyms or group genes in pathways see chart following*

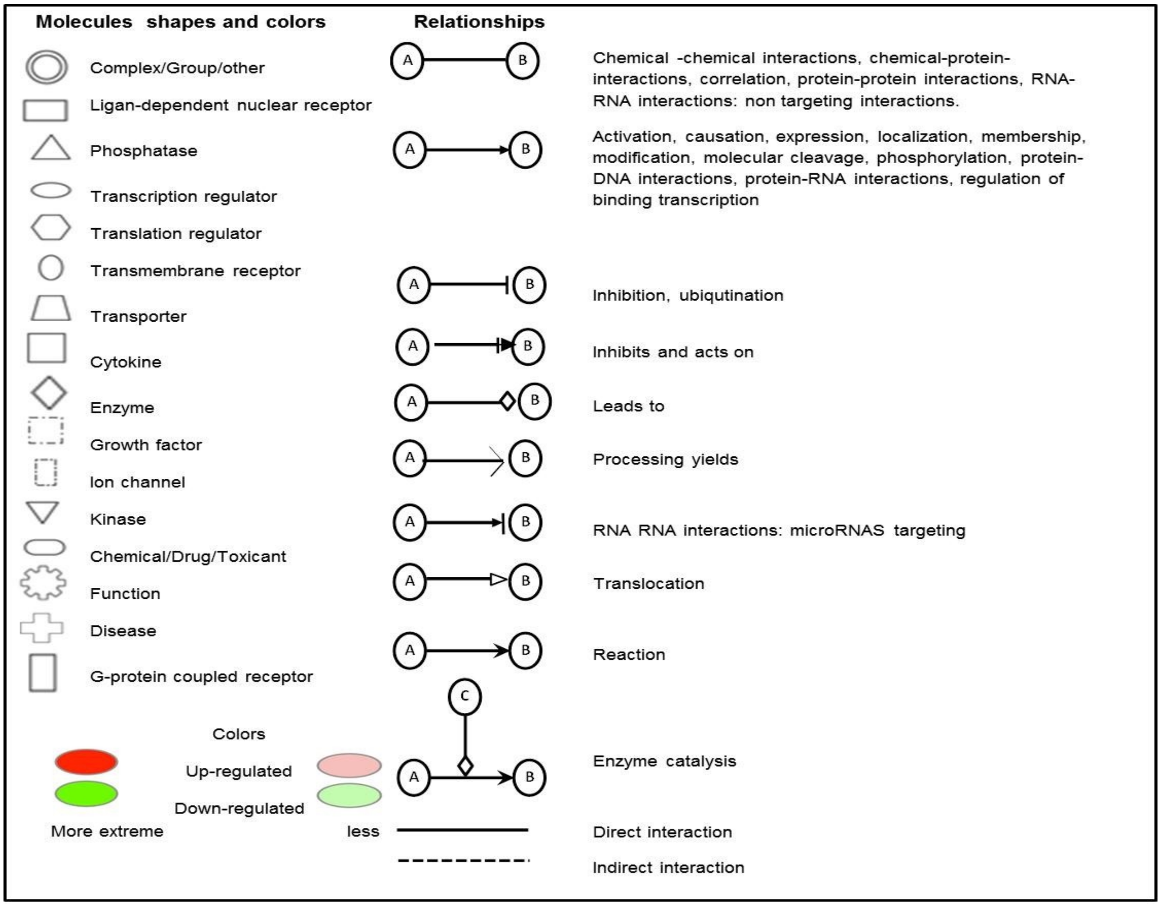
Molecules shapes and colors
Relationships
Complex/Group/other
Ligan-dependent nuclear receptor
Phosphatase
Transcription regulator
Translation regulator
Transmembrane receptor
Transporter
Cytokine
Enzyme
Growth factor
Ion channel
Kinase
Chemical/Drug/Toxicant
Function
Disease
G-protein coupled receptor
Colors
Up-regulated
Down-regulated
More extreme
less
A
B
C
Chemical -chemical interactions, chemical-protein-interactions, correlation, protein-protein interactions, RNA-RNA interactions: non targeting interactions.
Activation, causation, expression, localization, membership, modification, molecular cleavage, phosphorylation, protein-DNA interactions, protein-RNA interactions, regulation of binding transcription
Inhibition, ubiqutination
Inhibits and acts on
Leads to
Processing yields
RNA RNA interactions: microRNAS targeting
Translocation
Reaction
Enzyme catalysis
Direct interaction
Indirect interaction

Genes with synonyms or group genes in pathways IPA

Name gen	Name gen image pathways IPA
All RPS	40S ribosomal subunit
All RPL	60S ribosomal subunit
APH1A	γ-secretase
BACE2	β-secretase
BMPR2	GFR
CDKN1B	p27Kip1
CYC1	CYT C or complex III
DDIT4	REDD1
EIF1	A121
E2F4	E2F
EIF1, EIF2B2 and EIF2B5	eIF2
EIF2AK1	HRI
EIF2B2 and EIF2B5	eIF2β
EIF3B, EIF3C, EIF3D, EIF3G, EIF3I, EIF3K and EIF3L	eIF3
EIF4A1, EIF4A2 and EIF4A3	eIF4A
EIF4EBP1	4EBP or 4E-BP1 or eIF4BP
EIF4G1	eIF4γ and eIF4G
FAU	RPS30 of 40S ribosomal subunit
INPPL1	SHIP
ITGA2	Integrin
ITGB6 and ITGB5	β-Integrin
LMNA	Lamin A
MAP2K4	MKK4 or JNK
MCRS1	MSP58
PA2G4	EBP1
PARK7	DJ-1
PIK3CA	PI3K
PPP2R4 and PPP2R1A	PP2A
RND3	RHO
RPS6KA3	RSK
TXN2	TRX2

A

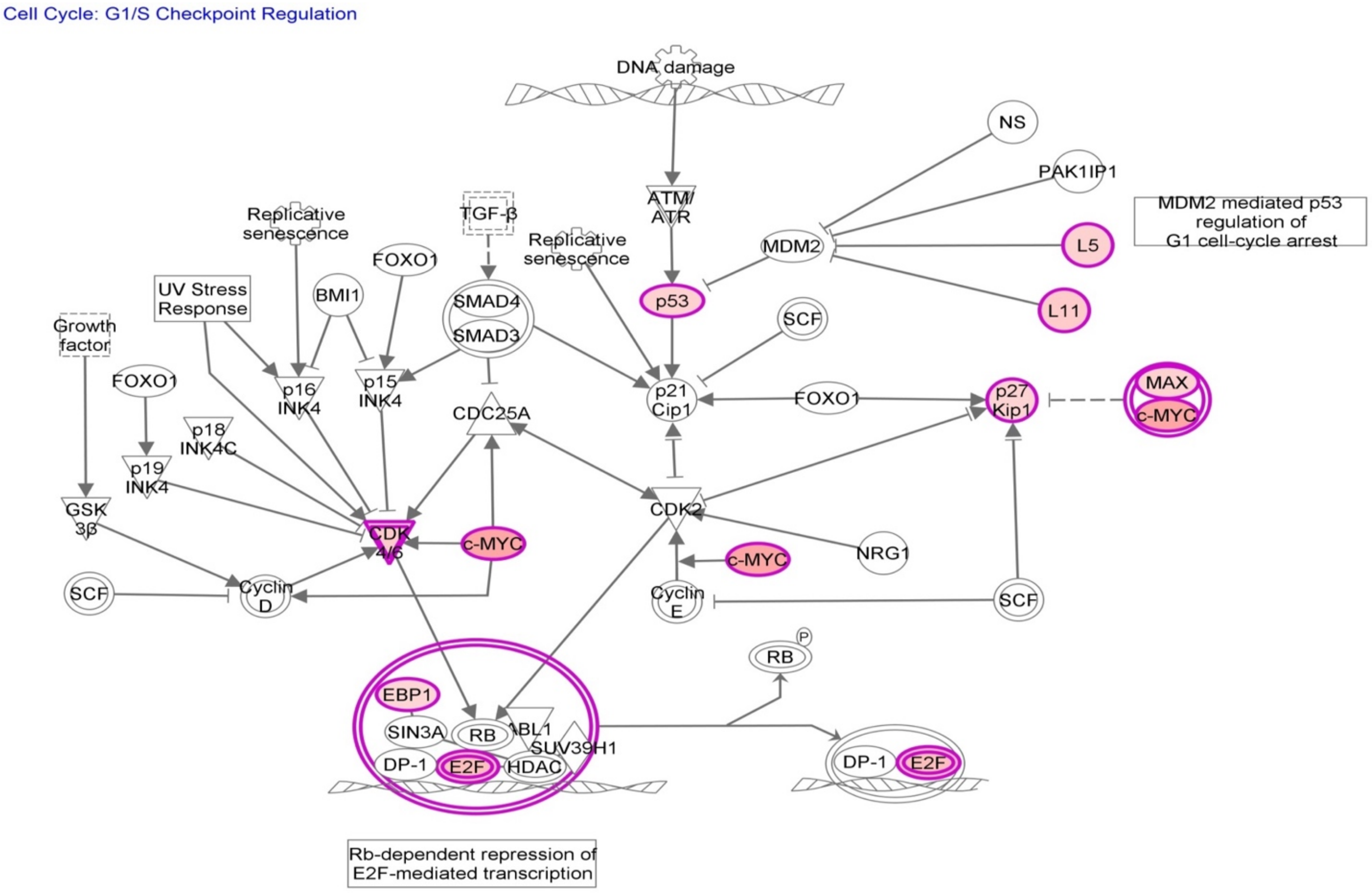

© 2000-2015 QIAGEN. All rights reserved.

B

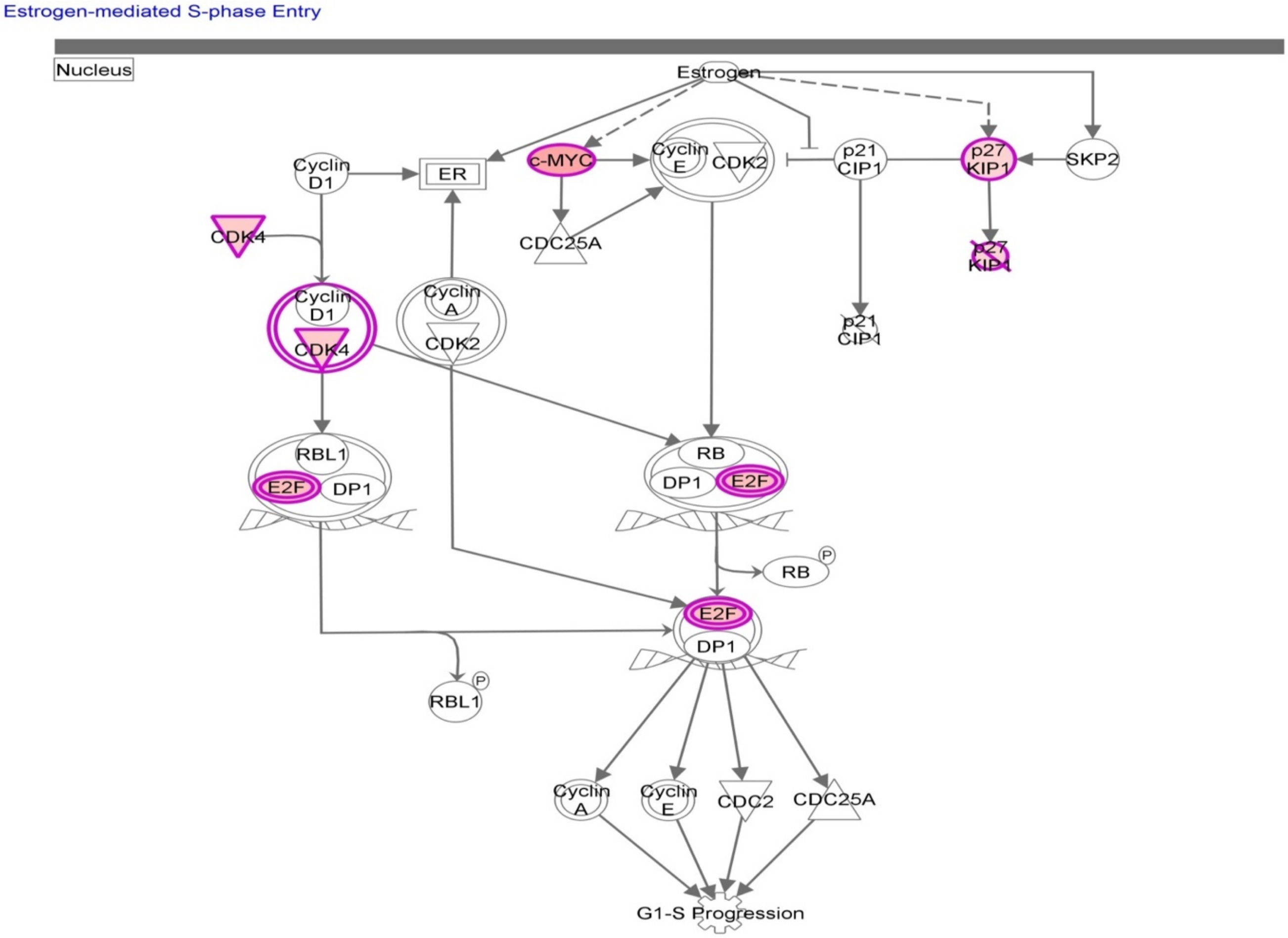

© 2000-2015 QIAGEN. All rights reserved.

C

Cell Cycle Regulation by BTG Family Proteins

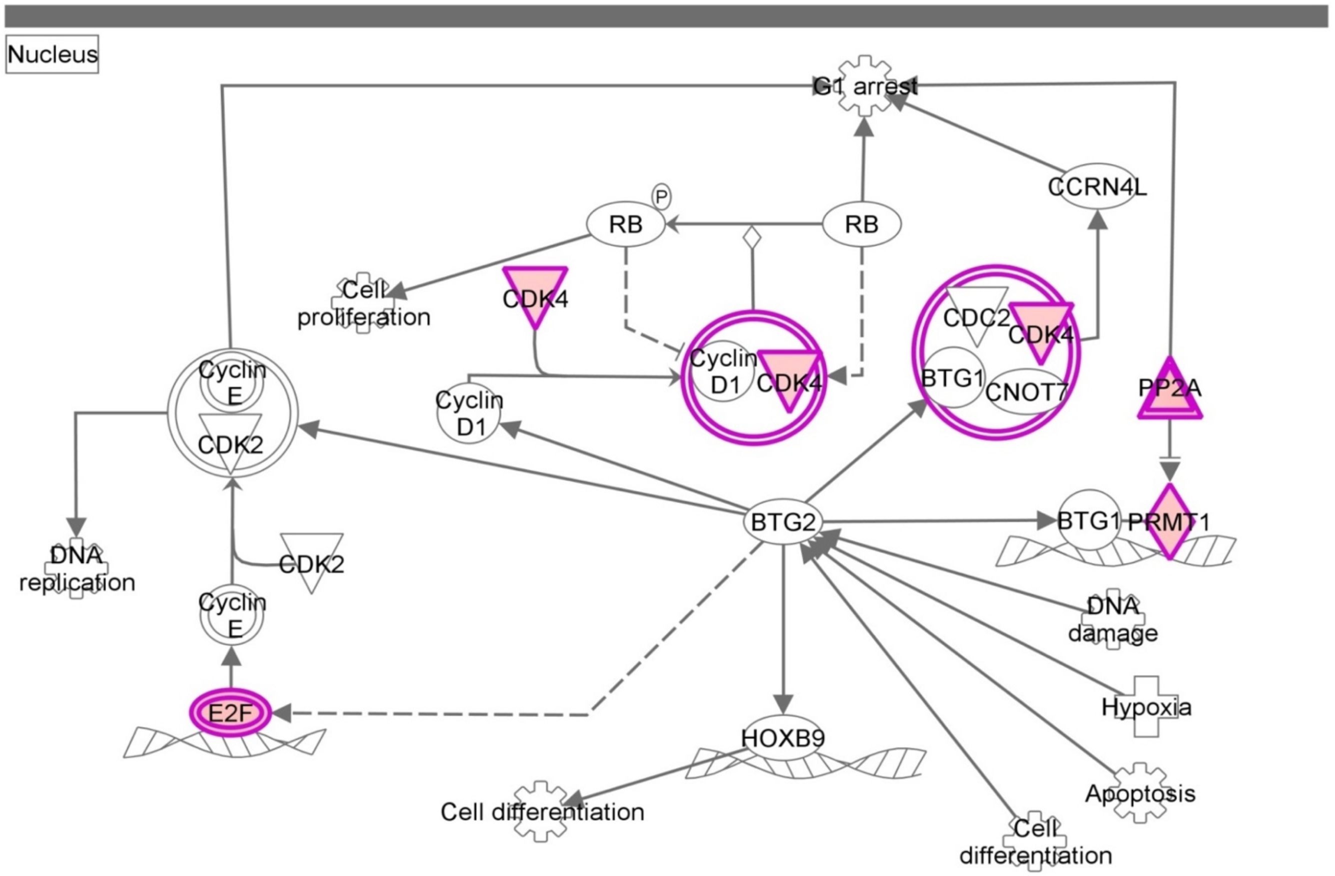

© 2000-2015 QIAGEN. All rights reserved.

D

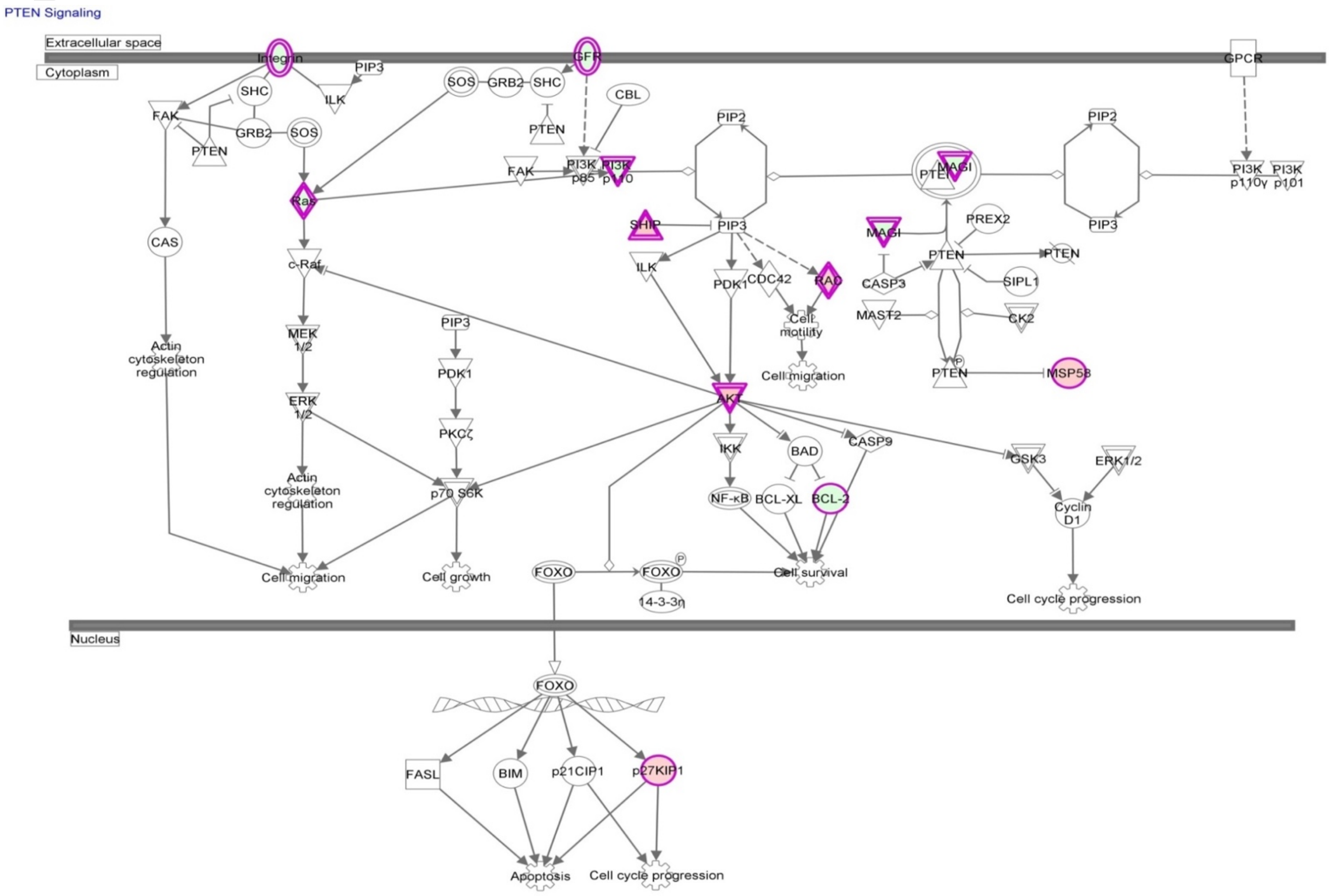

© 2000-2015 QIAGEN. All rights reserved.

E

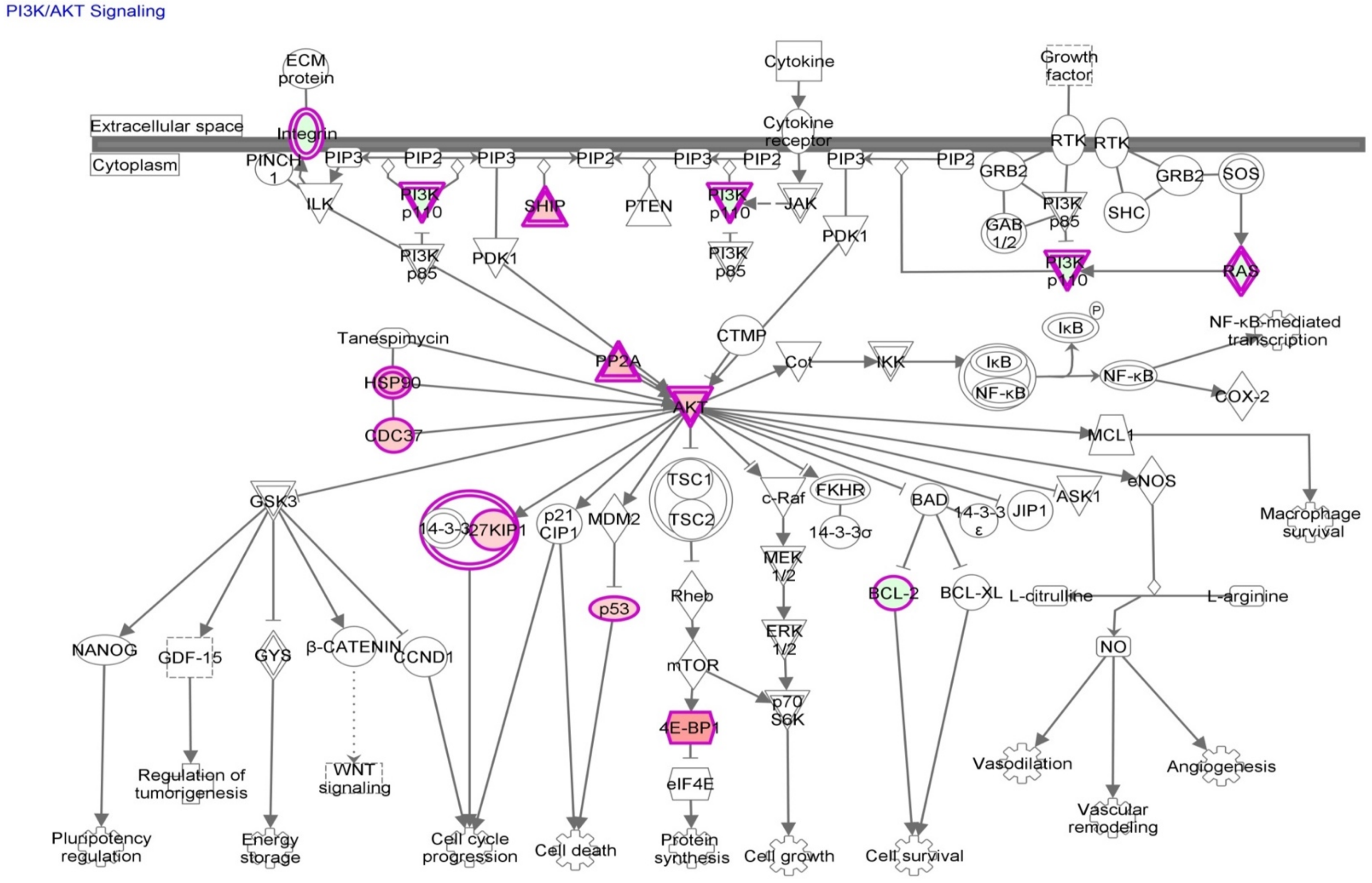

© 2000-2015 QIAGEN. All rights reserved.

F

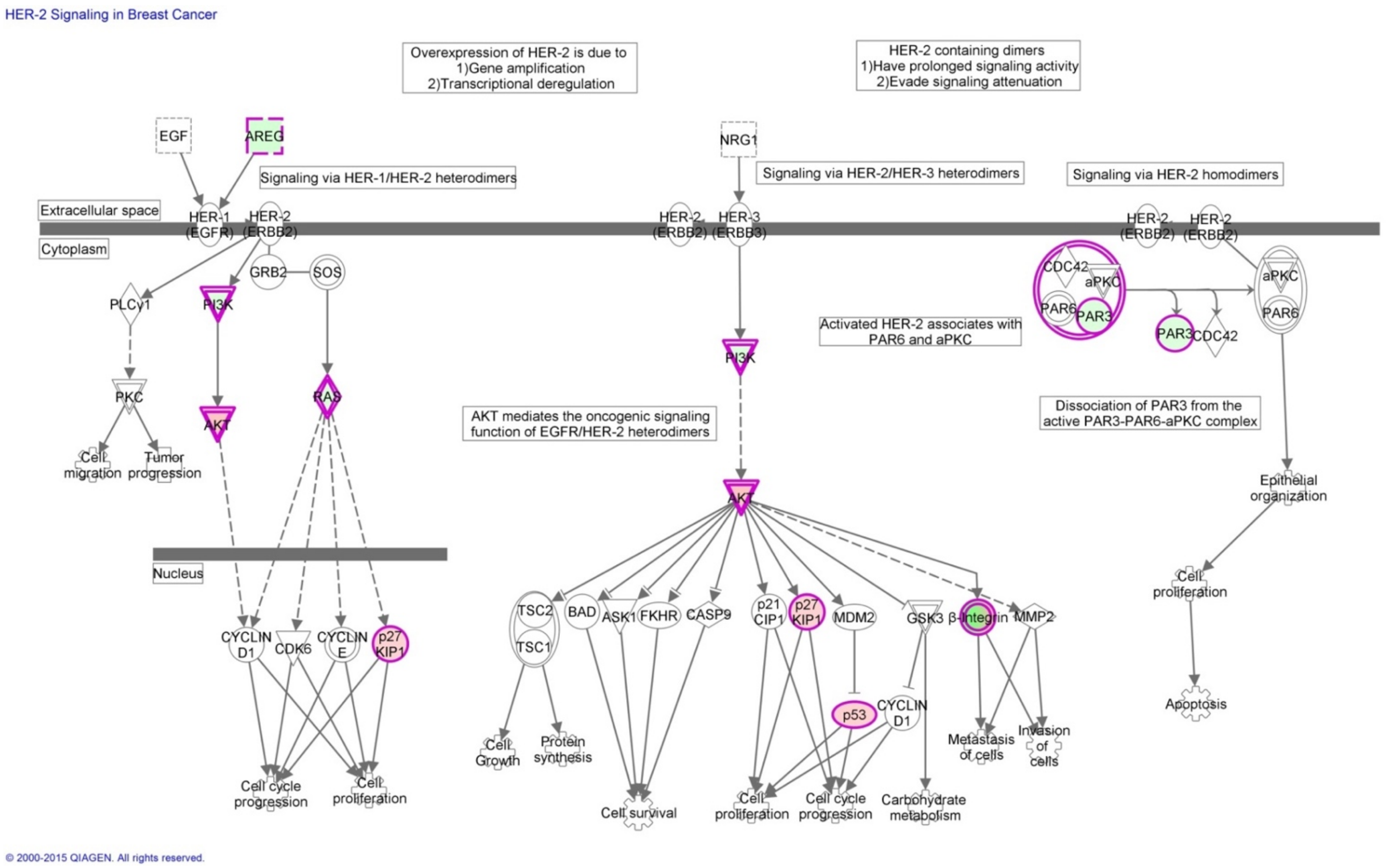

.

Supplementary Figure S3. Some interesting pathways associated with the cell cycle (IPA). (A) cell cycle G1/S checkpoint regulation, (B) Estrogen-mediated S-phase entry, (C) cell cycle regulation by BTG family protein, (D) PTEN signaling, (E) PI3K/AKT signaling and (F) HER-2 signaling in breast cancer

A

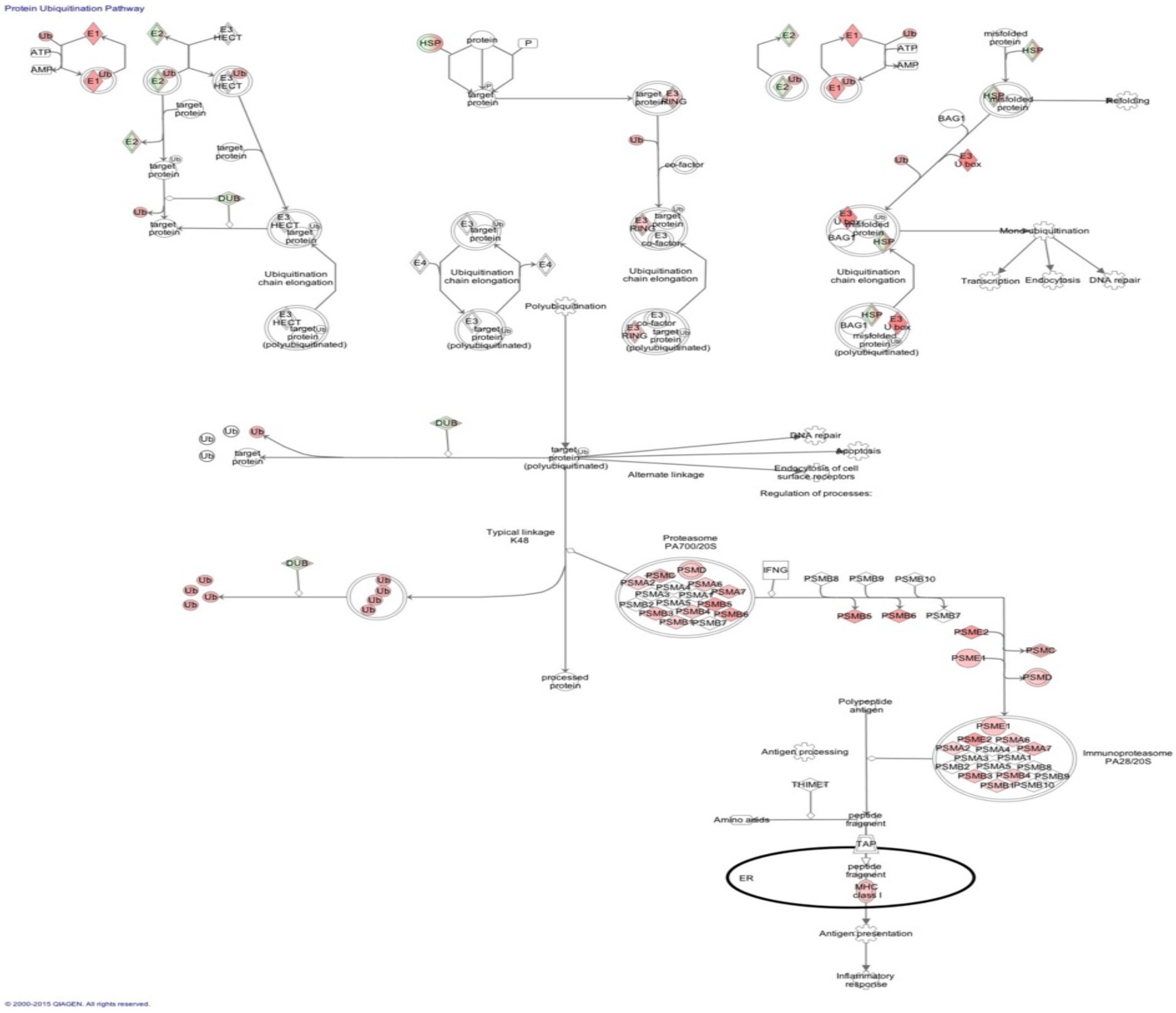

Protein Ubiquitination Pathway
E1
Ub
ATP
AMP
E2
E3 HECT
target protein
DUB
Ubiquitination chain elongation
E3 HECT target protein (polyubiquitinated)
HSP
protein
P
E3 RING
co-factor
E4
Polyubiquitination
misfolded protein
Refolding
BAG1
E3 U box
Mono-ubiquitination
Transcription
Endocytosis
DNA repair
target protein (polyubiquitinated)
Apoptosis
Endocytosis of cell surface receptors
Alternate linkage
Regulation of processes:
Typical linkage K48
Proteasome PA700/20S
PSMD
PSMC
PSMA1
PSMA2
PSMA3
PSMA4
PSMA5
PSMA6
PSMA7
PSMB1
PSMB2
PSMB3
PSMB4
PSMB5
PSMB6
PSMB7
IFNG
PSMB8
PSMB9
PSMB10
PSME2
PSME1
processed protein
Polypeptide antigen
Antigen processing
Immunoproteasome PA28/20S
THIMET
Amino acids
peptide fragment
TAP
ER
MHC class I
Antigen presentation
Inflammatory response
© 2000-2015 QIAGEN. All rights reserved.

B

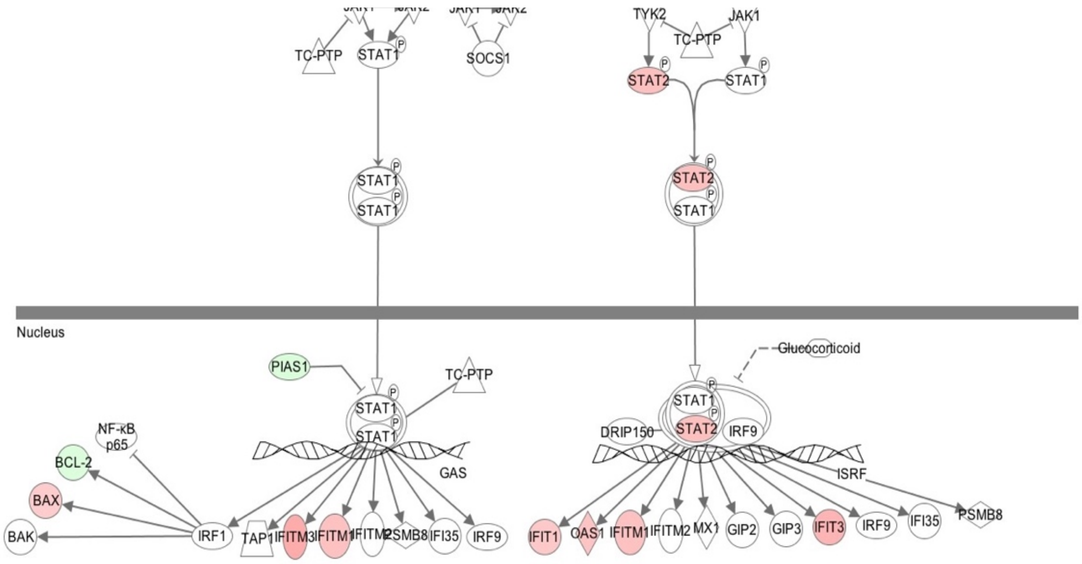

© 2000-2015 QIAGEN. All rights reserved.

C

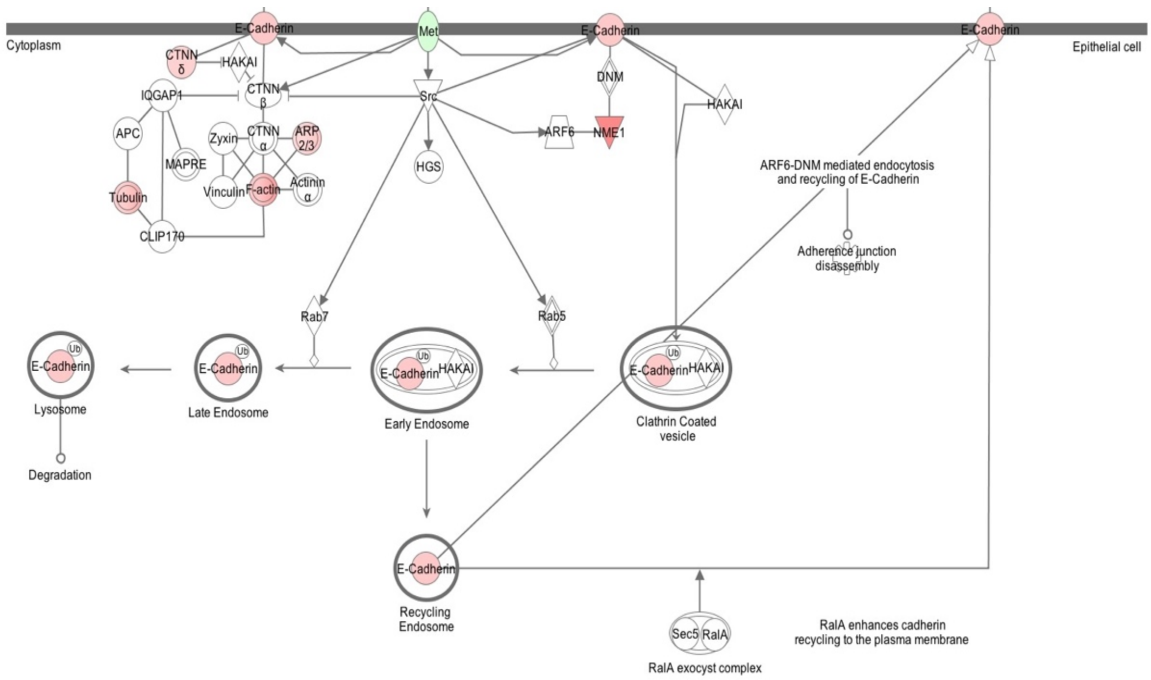

© 2000-2015 QIAGEN. All rights reserved.

D

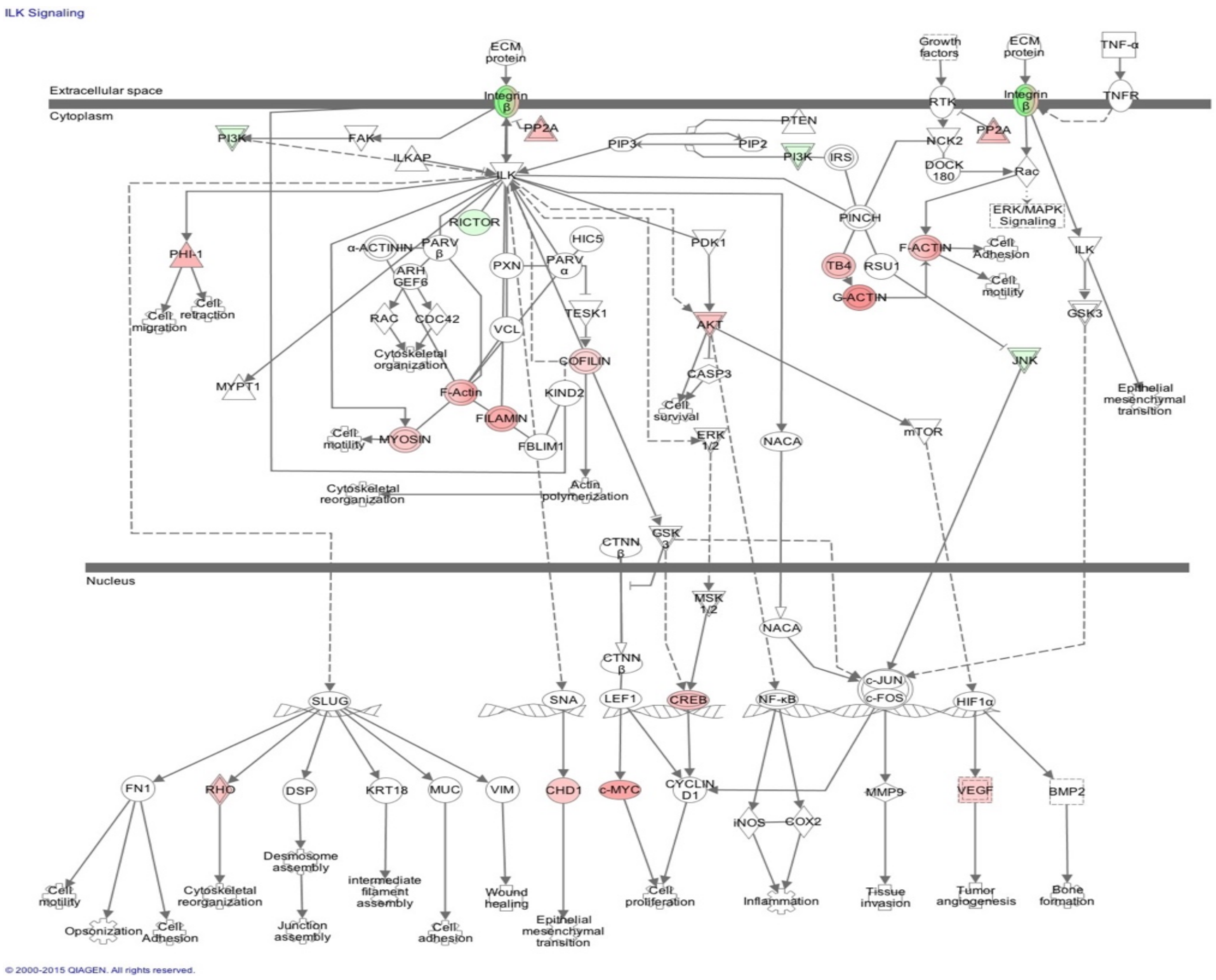
ILK Signaling
Extracellular space
Cytoplasm
Nucleus
ECM protein
Integrin β
PP2A
PI3K
FAK
ILKAP
ILK
PIP3
PIP2
PTEN
IRS
Growth factors
RTK
NCK2
DOCK 180
Rac
ERK/MAPK Signaling
TNF-α
TNFR
PINCH
TB4
RSU1
G-ACTIN
F-ACTIN
Cell Adhesion
Cell motility
GSK3
JNK
Epithelial mesenchymal transition
PHI-1
Cell migration
Cell retraction
MYPT1
RICTOR
α-ACTININ
PARV β
ARH GEF6
RAC
CDC42
Cytoskeletal organization
PXN
PARV α
HIC5
TESK1
VCL
COFILIN
F-Actin
FILAMIN
MYOSIN
KIND2
FBLIM1
Cytoskeletal reorganization
Actin polymerization
PDK1
AKT
CASP3
Cell survival
ERK 1/2
NACA
mTOR
CTNN β
GSK 3
MSK 1/2
SLUG
SNA
LEF1
CREB
NF-κB
c-JUN
c-FOS
HIF1α
FN1
RHO
DSP
KRT18
MUC
VIM
CHD1
c-MYC
CYCLIN D1
iNOS
COX2
MMP9
VEGF
BMP2
Opsonization
Desmosome assembly
Junction assembly
intermediate filament assembly
Cell adhesion
Wound healing
Cell proliferation
Inflammation
Tissue invasion
Tumor angiogenesis
Bone formation
© 2000-2015 QIAGEN. All rights reserved.

E

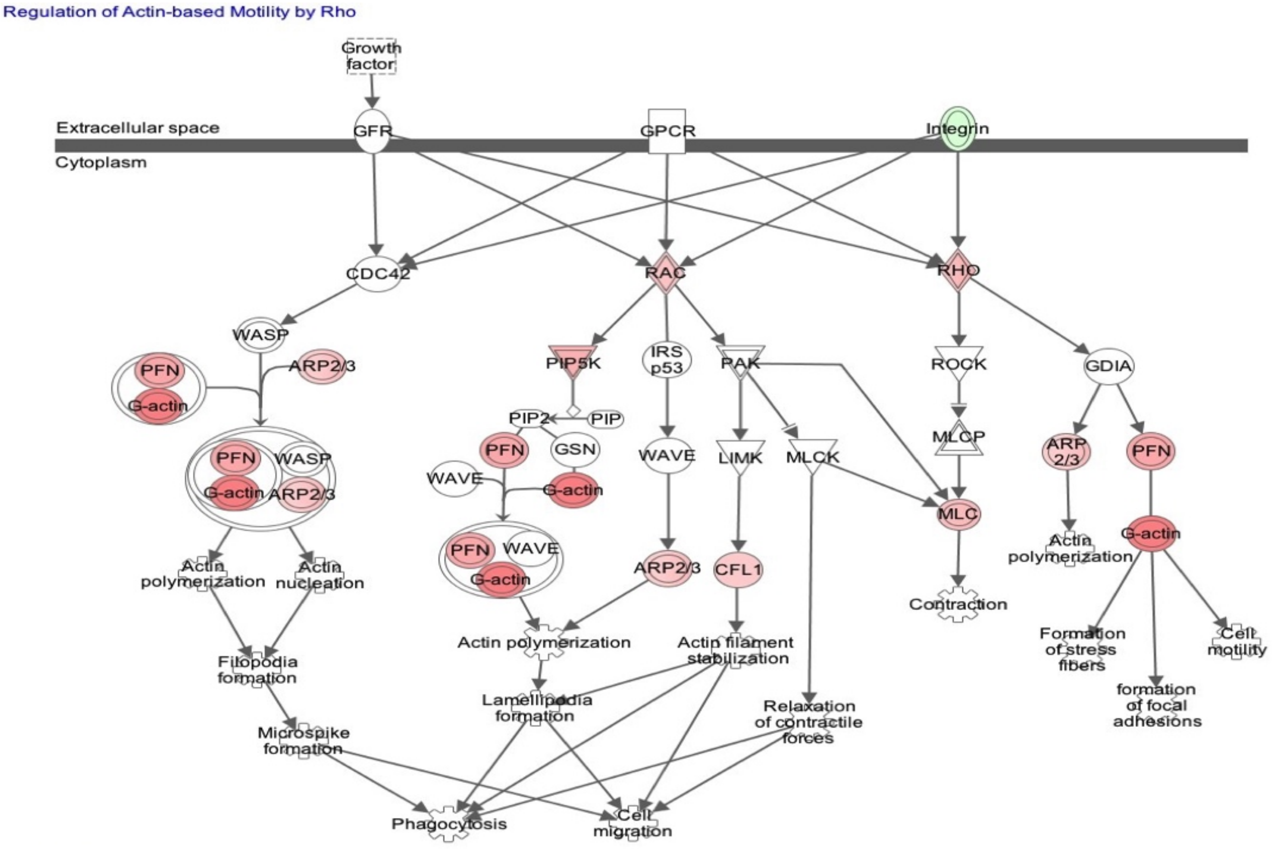

© 2000-2015 QIAGEN. All rights reserved.

F

Estrogen Receptor Signaling

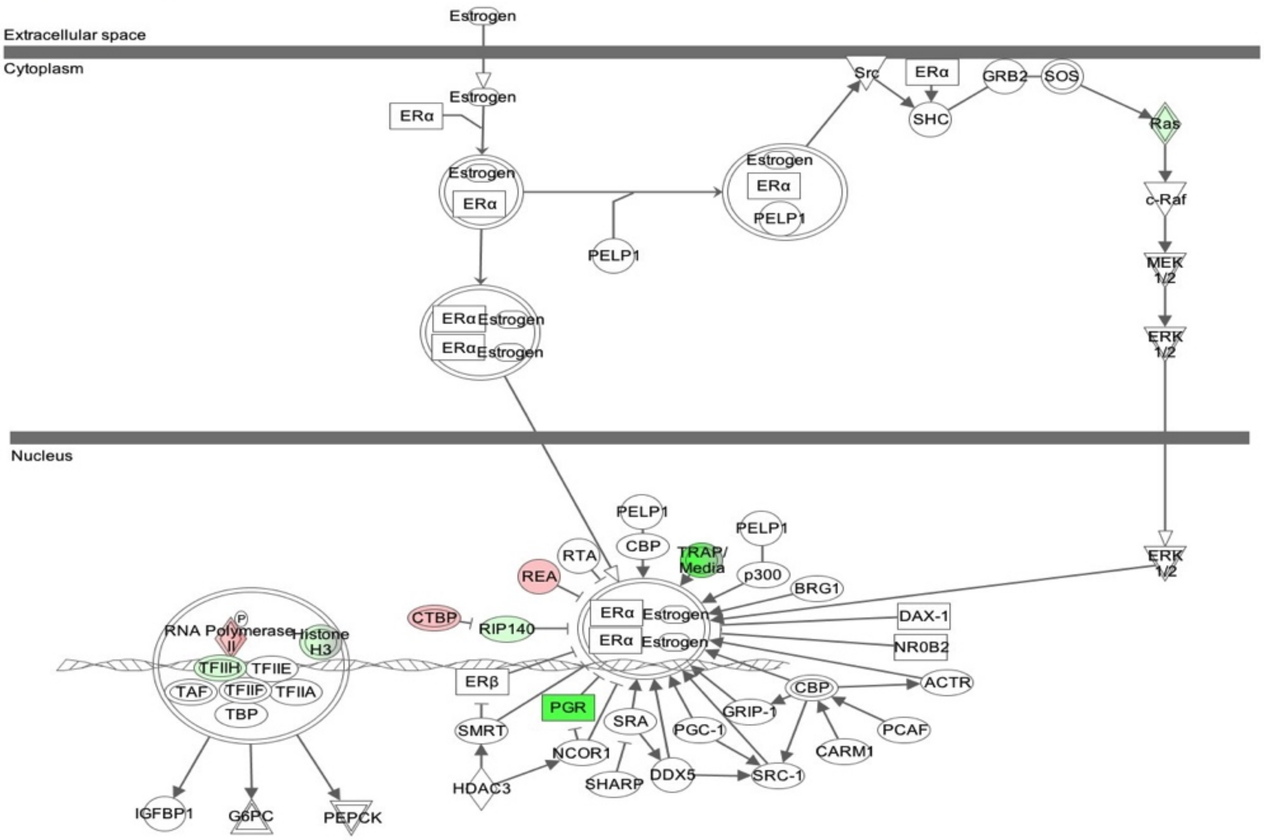

© 2000-2015 QIAGEN. All rights reserved.

G

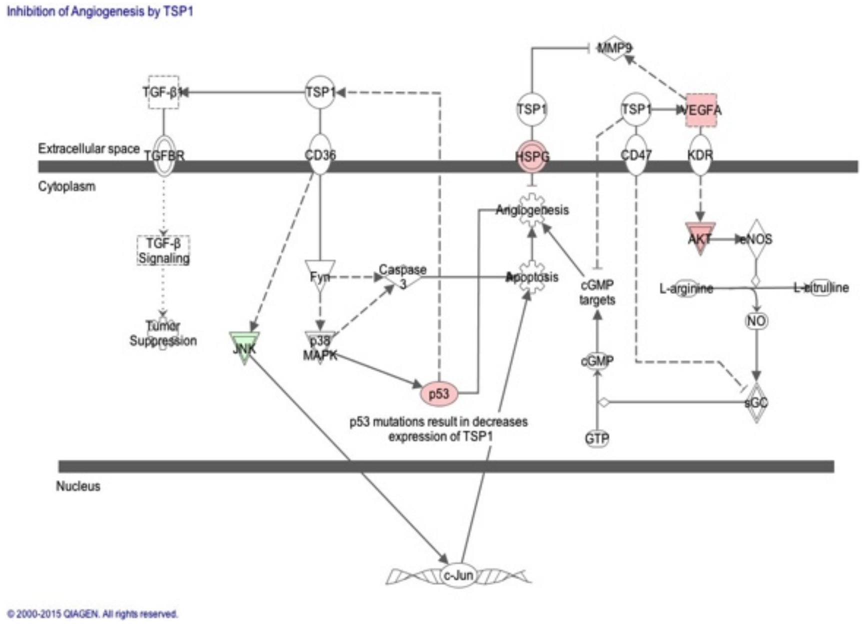
Inhibition of Angiogenesis by TSP1
MMP9
TGF-β1
TSP1
TSP1
TSP1
VEGFA
Extracellular space
TGFBR
CD36
HSPG
CD47
KDR
Cytoplasm
Angiogenesis
TGF-β Signaling
AKT
eNOS
Fyn
Caspase 3
Apoptosis
cGMP targets
L-arginine
L-citrulline
NO
Tumor Suppression
JNK
p38 MAPK
cGMP
p53
sGC
p53 mutations result in decreases expression of TSP1
GTP
Nucleus
c-Jun
© 2000-2015 QIAGEN. All rights reserved.

H

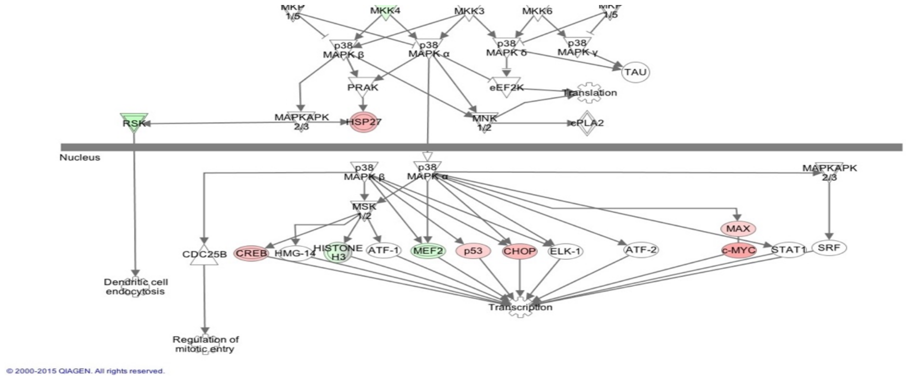
MKK4
MKK3
MKK6
p38 MAPK β
p38 MAPK α
p38 MAPK δ
p38 MAPK γ
TAU
PRAK
eEF2K
Translation
RSK
MAPKAPK 2/3
HSP27
MNK 1/2
cPLA2
Nucleus
p38 MAPK β
p38 MAPK α
MAPKAPK 2/3
MSK 1/2
MAX
CDC25B
CREB
HMG-14
HISTONE H3
ATF-1
MEF2
p53
CHOP
ELK-1
ATF-2
c-MYC
STAT1
SRF
Dendritic cell endocytosis
Transcription
Regulation of mitotic entry
© 2000-2015 QIAGEN. All rights reserved.

Supplementary Figure S4. Some canonical pathways associated with other cellular processes are also; (A) protein ubiquitination, (B) Interferon signaling, (C) Remodeling of epithelial adherents junctions, (D) ILK signaling, (E) Integrin signaling, (F)) Estrogen receptor signaling, (G)Inhibition of angiogenesis by TSP1 and (H) p38 MAPK signaling.

A

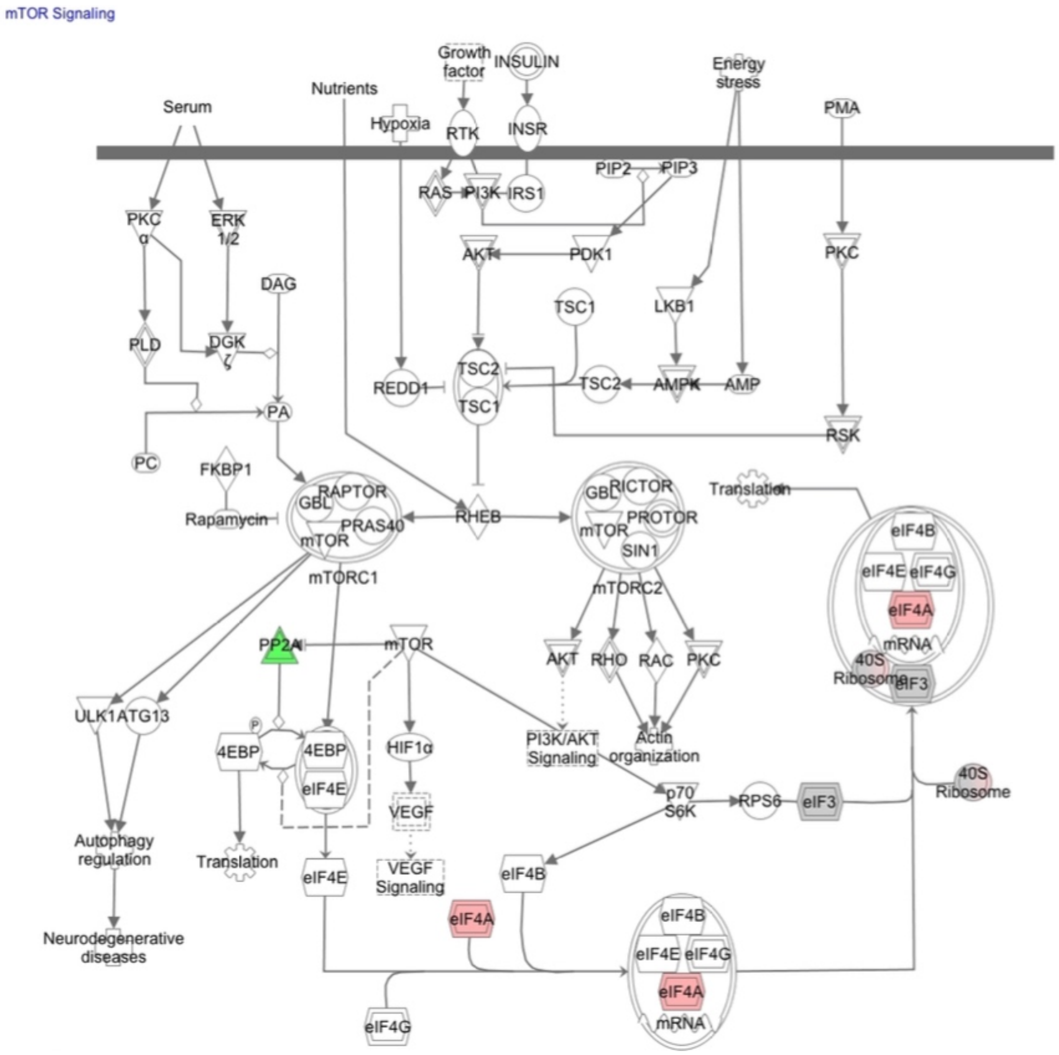
mTOR Signaling
Growth factor
INSULIN
Energy stress
Nutrients
Serum
Hypoxia
RTK
INSR
PMA
PIP2
PIP3
RAS
PI3K
IRS1
PKC α
ERK 1/2
AKT
PDK1
PKC
DAG
TSC1
LKB1
PLD
DGK ζ
TSC2
REDD1
TSC1
TSC2
AMPK
AMP
PA
RSK
PC
FKBP1
Rapamycin
GBL
RAPTOR
PRAS40
mTOR
mTORC1
RHEB
GBL
RICTOR
PROTOR
mTOR
SIN1
mTORC2
Translation
eIF4B
eIF4E
eIF4G
eIF4A
mRNA
40S Ribosome
eIF3
PP2A
mTOR
AKT
RHO
RAC
PKC
ULK1
ATG13
4EBP
4EBP
eIF4E
HIF1α
PI3K/AKT Signaling
Actin organization
40S Ribosome
VEGF
p70 S6K
RPS6
eIF3
Autophagy regulation
Translation
eIF4E
VEGF Signaling
eIF4B
eIF4A
eIF4B
eIF4E
eIF4G
eIF4A
mRNA
Neurodegenerative diseases
eIF4G

B

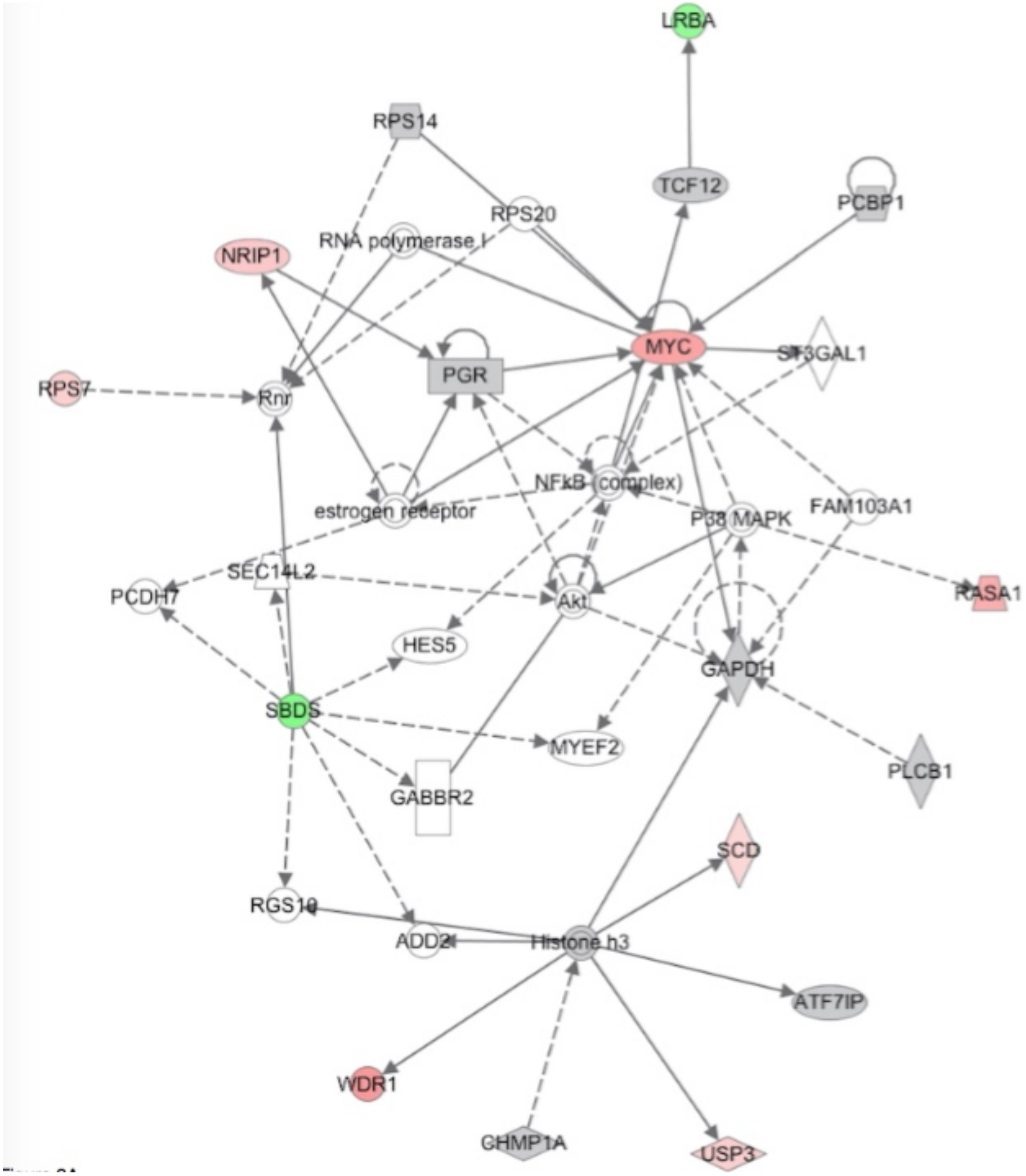

LRBA
RPS14
TCF12
PCBP1
RPS20
RNA polymerase I
NRIP1
MYC
ST3GAL1
PGR
RPS7
Rnr
NFkB (complex)
estrogen receptor
FAM103A1
P38 MAPK
SEC14L2
PCDH7
Akt
RASA1
HES5
GAPDH
SBDS
MYEF2
PLCB1
GABBR2
SCD
RGS10
ADD2
Histone h3
ATF7IP
WDR1
CHMP1A
USP3

C

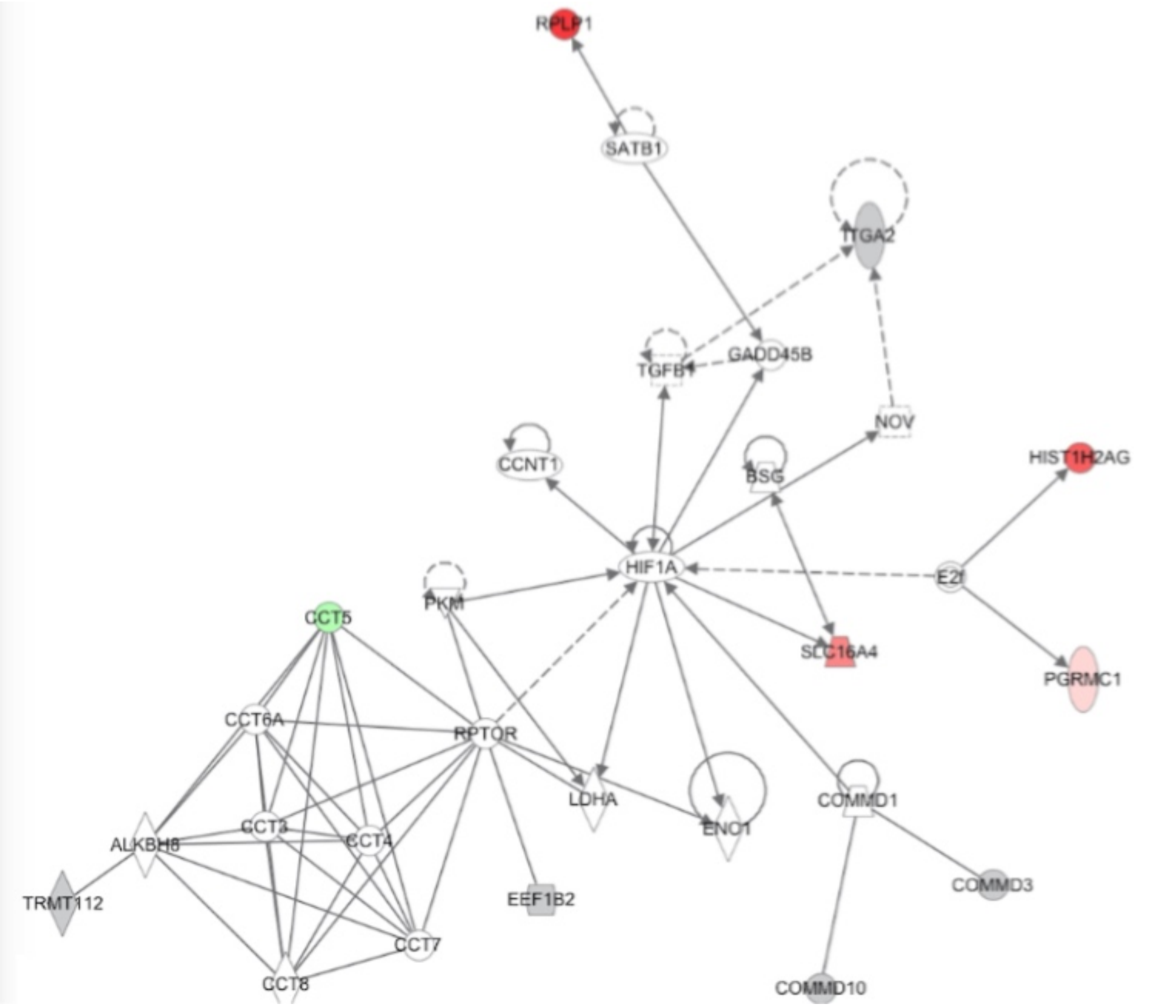

Fig S5. Dysregulated pathways analysis using 63 lncRNA that present his neighbor RNAm dysregulated. A) mTOR network. B) MYC Network and C) HIF network.

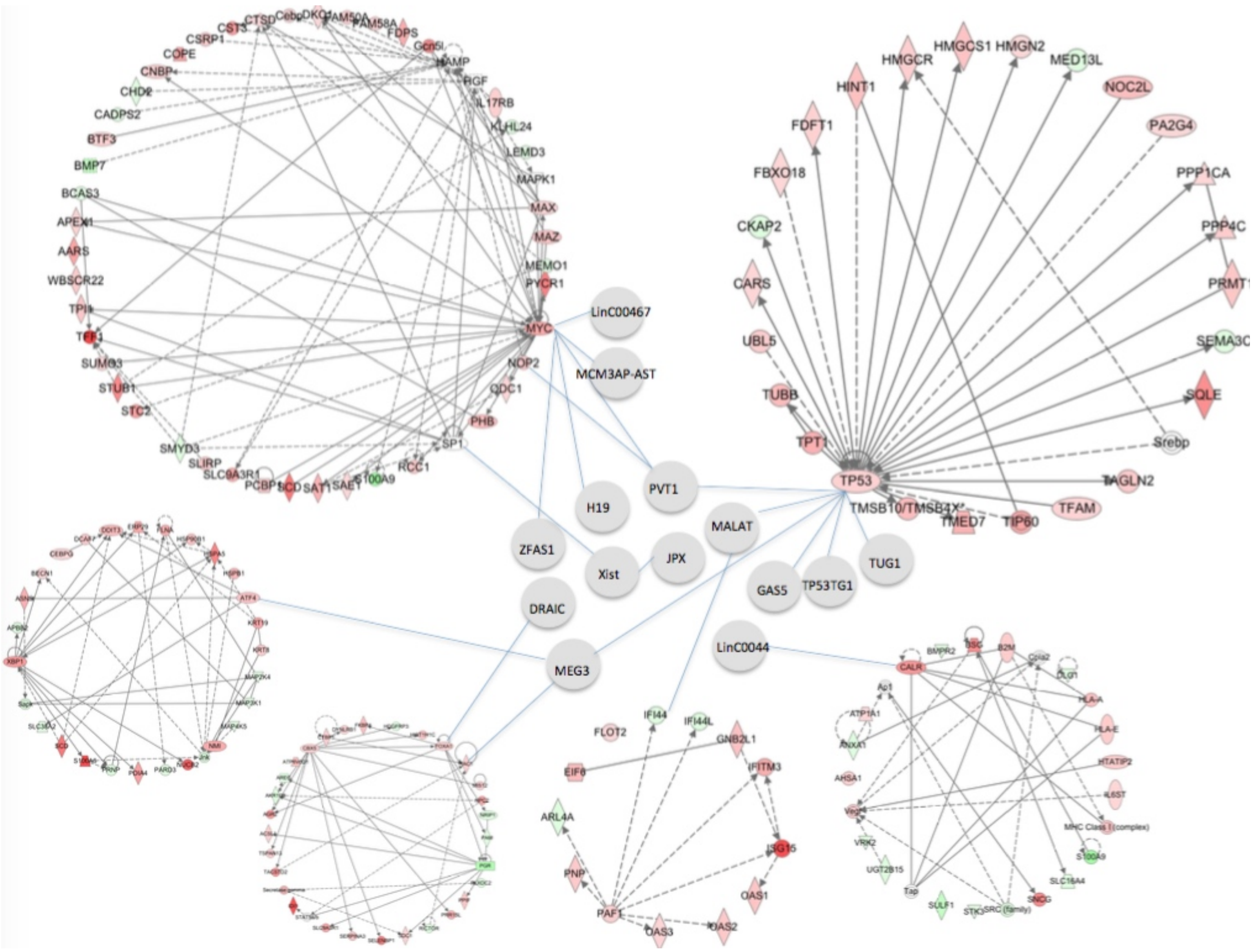

Supplementary Figure S6. Correlation between mRNA/lncRNAs networks. Green and red circles represent the mRNAs and gray circles the lncRNAs .
